# Male reproductive biology was reshaped during placental mammal diversification through epididymal secretome expansion

**DOI:** 10.1038/s44319-026-00817-1

**Published:** 2026-06-03

**Authors:** Jose M Ranz, Alberto Civetta

**Affiliations:** 1 Department of Ecology and Evolutionary Biology, University of California Irvine, Irvine, CA USA; 2 Department of Systems Biology, University of California Irvine, Irvine, CA USA; 3 Department of Biology, University of Winnipeg, Winnipeg, MB Canada

**Keywords:** Chromatin, Transcription & Genomics, Evolution & Ecology

## Abstract

The evolutionary timing of the origin of secretory proteins underlying post-mating reproductive processes remains uncharacterized in vertebrates. We dated the origin of 2520 human tissue-specific genes encoding secretory proteins across vertebrate evolution, finding that the male reproductive (MR) secretome underwent a dramatic expansion during the eutherian (placental mammal) diversification, experiencing a 6.8-fold gene gain—the largest increase compared to any other secretome or transition in vertebrate evolution. These genes are predominantly expressed in the epididymis, where they protect sperm and drive sperm maturation, influencing essential post-mating reproductive processes and male reproductive outcome. In contrast, MR secretome genes that originated along other evolutionary branches are primarily associated with sperm structure, motility, egg binding, and fusion. These findings provide molecular evidence for a major reconfiguration of male reproductive biology during placental mammal diversification through the MR secretome, affecting primarily the epididymis.

## Synopsis

The male reproductive secretome was largely reshaped during eutherian radiation, primarily via epididymis-expressed genes with unique functional traits compared to male reproductive secretome genes originated from other evolutionary stages.

- Systematic mapping of branch-of-origin for all secretome-encoding genes across the vertebrate phylogeny.
- The male reproductive (MR) secretome underwent the largest gene gain during the eutherian radiation compared to any other secretome and evolutionary transition.
- Most of the new genes impacting the MR secretome during the eutherian radiation are more likely to be expressed in the epididymis than in testis.
- The MR secretome genes originating during the eutherian radiation showed enrichment for a more diverse immune repertoire and distinct proteolytic processes compared to other MR secretome genes.

## Abstract (web summary)

The male reproductive secretome was largely reshaped during eutherian radiation, primarily via epididymis-expressed genes with unique functional traits compared to male reproductive secretome genes originated from other evolutionary stages.

## Graphical abstract

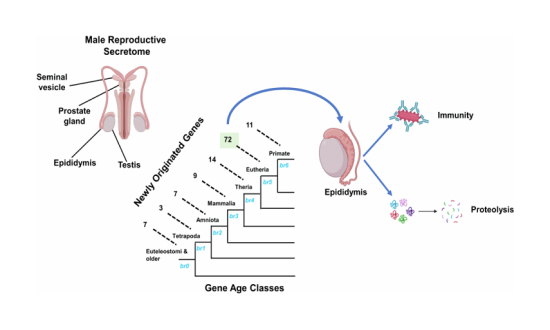

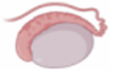

## Introduction

In eukaryotes, a portion of proteins—including channels, hormones, peptidases, and receptors—is secreted extracellularly. These secreted proteins represent the largest fraction of tissue-specific proteomes (Kelly, 1985; Uhlen et al, 2015). In humans, the proper manufacturing and composition of tissue-specific secretomes are indicators of healthy physiology and development (Houerbi et al, 2024; Wang and Kaufman, 2016). Through expression and computational approaches (Uhlen et al, 2015; Uhlen et al, 2019), tissue-specific secretomes have been characterized, including those associated with male and female reproductive organs, which manufacture the germline and facilitate successful fertilization, thus being essential for species perpetuation. For example, the male reproductive secretome includes proteins that protect sperm, guide their development and maturation, and accompany them to the female reproductive tract (Uhlen et al, 2019).

Recently, a subset of genes encoding secreted proteins in the male reproductive system of *D. melanogaster*, known as Seminal Fluid Proteins (SFPs), has been shown to have functional and evolutionary properties that depend on when they originated during the Diptera radiation (Ranz et al, 2025). Notably, a sizable fraction of SFP-encoding genes is evolutionarily ancient. This conflicts with the notion that sex-related genes feature high rates of evolutionary change (Patlar et al, 2021; Zhang et al, 2007), particularly those associated with male biology, as males are the preferential target of sexual selection (Darwin, 1871). This finding raises several questions: (i) Is the male reproductive secretome phylogenetically different in evolutionary age overall, and younger in particular, relative to other secretomes? (ii) Is the buildup of the male reproductive secretome gradual or punctuated by bursts of origin at specific phylogenetic branches? (iii) If such bursts have occurred, are they related to changes in male reproductive biology preferentially associated with particular gene functions and tissues?

## Results and discussion

**Figure 1:**
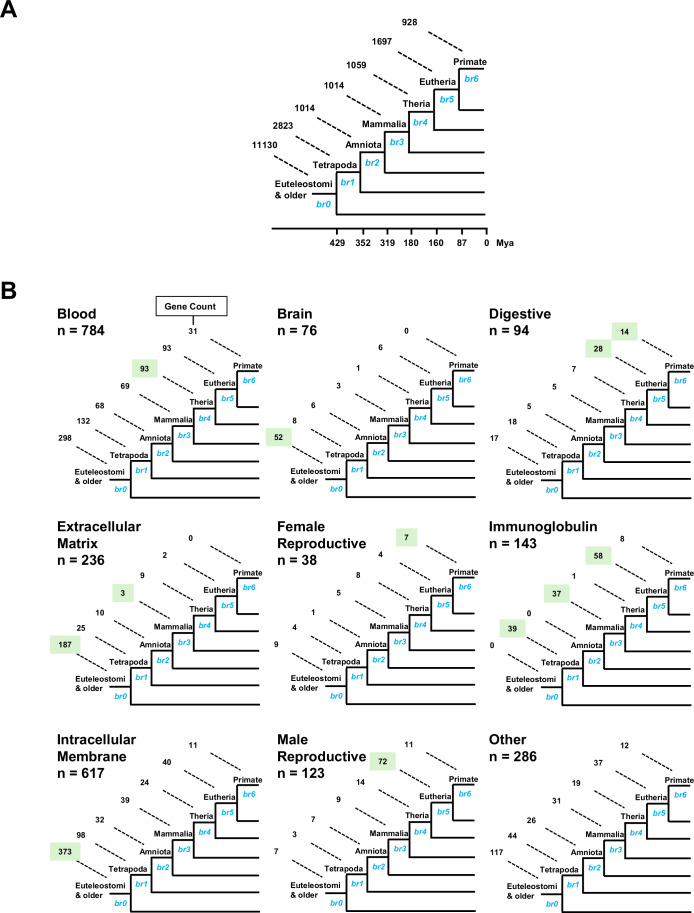
Phylogenetic origin of secretome-encoding genes in the human genome across the vertebrate phylogeny. (**A**) Human protein-coding genes inferred to be present in the euteleostomi genome and subsequently added during key evolutionary transitions in vertebrates. Seven phylogenetic branches were considered: euteleostomi and older (*br0*), tetrapoda (*br1*), amniota (*br2*), mammalia (*br3*), theria (*br4*), eutheria (*br5*), and primates (*br6*). The number of genes inferred to be present in the genome of species associated with each phylogenetic branch is indicated (Chen et al, 2025; Shao et al, 2019). Absolute estimates of divergence times in million years were obtained from TimeTree 5 (Kumar et al, 2022). (**B**) Phylogenetic distribution of protein-coding genes that are part of different secretomes (Chen et al, 2025; Shao et al, 2019). The number of genes originated (i.e., gained relative to those existing in the immediately older branch) is indicated for each branch. Data for coding genes with unknown tissue origin can be found in Dataset EV1. Green shaded counts represent cases of statistical enrichment at 1% FDR, according to a post hoc residual analysis following a *χ*^2^ test of independence (Appendix Table S1). Source data are available online for this figure.

To address the above questions, we focused on humans for two reasons. First, humans possess the most comprehensively characterized collection of secretomes at the molecular level (Uhlen et al, 2019). The human secretome comprises 2520 genes encoding predicted secretory proteins based on subcellular location, presence of signal peptides, and absence of transmembrane domains. These secreted proteins are categorized into nine distinct secretomes based on where they are secreted in the human body (Methods). Second, the evolutionary origin of 19,665 protein-coding human genes has been reliably inferred across the vertebrate phylogeny, distinguishing evolutionary branches that correspond to key evolutionary transitions. This was accomplished using a combination of high-quality reciprocal genome-wide alignments that informed about the gene’s microsyntenic context, plus the phylogenetic distribution of orthologs (Chen et al, 2025). Accordingly, human genes were inferred to have originated at the time of euteleostomi or subsequently during the tetrapoda, amniota, mammalia, theria, eutheria, and primate radiations, respectively (Chen et al, 2025) (Fig. 1A).

We established the evolutionary origin of 2511 genes from the human secretome across the vertebrate phylogeny and tested for possible bursts of gene origin across secretomes and phylogenetic branches (Fig. 1B; Dataset EV1). The number of genes originated across the seven branches by secretome combinations departed from random expectation (*χ*^2^ test of independence = 843.82, *P* < 2.2 × 10^−16^). Post hoc tests revealed deviations in six branches across eight secretomes at 1% FDR, including 12 secretome-by-branch combinations that experienced significant expansions (Appendix Table S1). A particularly dramatic expansion of 72 genes, which added to the male reproductive (MR) secretome, occurred at the time of the radiation of placental mammals (eutheria; i.e., branch 5) (Fig. 1B; Dataset EV1; Appendix Table S1). Although other substantial gene gains were observed for the immunoglobulin and digestive secretomes at this branch, as well as for extracellular matrix and intracellular membrane secretomes at branch 0, residual analysis indicated that these expansions were less pronounced than that of the MR secretome at branch 5 (Appendix Table S1).

**Figure 2:**
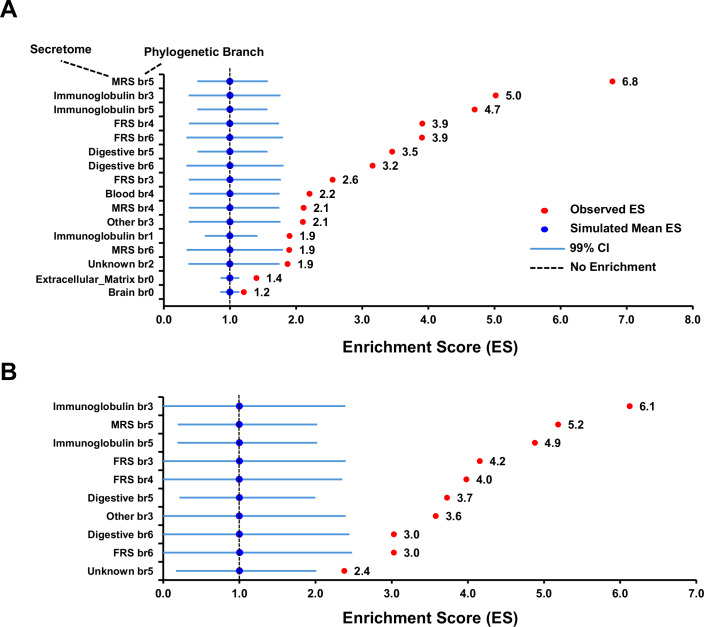
The male reproductive secretome added more genes during the eutherian radiation than any other secretome-by-branch combination during vertebrate evolution. (**A**) Forest plot showing enrichment scores (ESs; red dots) for the 16 secretome-by-phylogenetic-branch combinations statistically significant at 1% FDR. ES represents the fold gain of genes relative to random expectation. The departure of the observed ESs (red dots) from their 99% CI and simulated average (blue dots) according to Monte Carlo simulations (*n* = 100,000) can be appreciated. The simulated averages for ES values are virtually 1, i.e., indicating no enrichment (dashed vertical line). (**B**) Like panel (**A**), but after omitting secretome-encoding genes with high-confidence paralog predictions in the human genome as established with DIOPT v9.0 (Hu et al, 2011). Monte Carlo simulations (*n* = 100,000) were done identically to those in (**A**). In total, 856 secretome-encoding genes were considered. Ten secretome-by-phylogenetic-branch combinations are statistically significant at 1% FDR. MRS and FRS, male and female reproductive secretomes, respectively. Source data are available online for this figure.

We further calculated secretome expansion as the proportion of genes gained at each branch and secretome combination normalized by branch size, i.e., the fraction of genes originating at that branch relative to the total number of genes (“Methods”). The resulting enrichment scores calculated across secretomes and branches showed that the eutherian radiation branch had a 6.8-fold higher gain of new genes for the MR secretome than expected under a uniform distribution proportional to gene number (“Methods”). Monte Carlo simulations (*n* = 100,000) indicated that an enrichment score equal to or higher than that of the MR secretome-branch 5 combination had a very low probability of occurring by chance alone (*P* < 1 × 10^−5^; 99% CI = 0.56–1.57). No other secretome-by-branch combination exhibited a fold gene gain as substantial as the one experienced by the MR secretome during the eutherian radiation (Fig. 2a). The probability that the observed enrichment was the maximum across all secretome-by-branch combinations by chance alone was also negligible (*P* = 5.93 × 10^−3^). Together, the two tests implemented consistently point to a substantial expansion of the MR secretome during the eutherian radiation, a pattern that we found to be largely independent from multigene family proliferations (Fig. 2B; Appendix Tables S1 and S3; Appendix).

**Figure 3:**
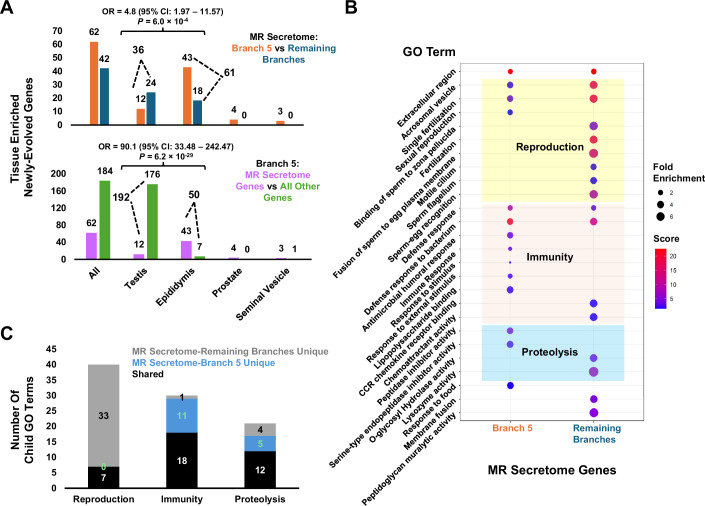
Genes contributing to the male reproductive (MR) secretome gained during the eutherian radiation are preferentially expressed in the epididymis and are relevant for biased reproductive outputs at the post-mating level. (**A**) Clustered column chart showing the presence of genes with enriched expression across the four male reproductive system tissues examined in the Human Protein Atlas (i.e., genes that feature at least fourfold higher expression in a given tissue compared to any other body tissue) between different sets of secretome-encoding genes. Top: MR secretome genes originated during the eutherian radiation (i.e., branch 5 in Fig. 1B) vs. MR secretome genes that originated in the other six evolutionary transitions (i.e., branches 0–4 and 6 in Fig. 1B). Bottom: MR secretome genes originated during the eutherian radiation vs. any other protein-encoding gene that originated in that same radiation. Two-tailed Fisher’s exact tests were performed to test for significantly different associations between preferential expression in testis vs. epididymis for the two subsets of genes compared in each plot. The odds ratio with its 95% confidence interval, plus the *P* value, is shown for each test. (**B**) Bubble charts showing gene ontology (GO) enrichment patterns based on a majority-based decision method using information from three tools (DAVID, PANGEA and PAN-GO) followed by removal of redundant terms using rrvgo (Supek et al, 2011). The size of the dots is proportional to the gene-class enrichment, while the color of the dots corresponds to the FDR-corrected *P* values. (**C**) Stacked column chart showing the main broad functional themes to which different sets of secretome-encoding genes contribute to the enrichment of parental GO terms. The number of child GO terms within parental terms is indicated, and whether associated only with the eutherian radiation, with any of the other six evolutionary transitions, or with both sets is color-coded. Source data are available online for this figure.

We then sought distinctive functional signatures associated with the 72 genes of the MR secretome that originated during the eutherian radiation and the remaining 51 genes across other phylogenetic branches. We first examined expression profiles. The Human Proteome Atlas categorized 104 of the genes as tissue-enriched, meaning they feature at least fourfold higher expression in a given tissue compared to any other body tissue. Of these, 36 were enriched in the testis, 61 in the epididymis, 4 in the prostate, and 3 in the seminal vesicles. Considering the counts of these four types of preferentially expressed genes in the eutherian branch versus the other branches, we tested for preferential association between the origin of new genes and specific tissues. We found a significant excess of epididymis-enriched genes that arose during the eutherian radiation relative to testis-enriched genes, with the latter being more tightly associated with the remaining six branches (*χ*^2^ test of independence = 18.07, *P* = 4.5 × 10^−4^; Appendix Table S2). This result reveals that during the eutherian radiation, the epididymis secretome was the most significantly reconfigured. Specifically, 69% (43/62) of the newly added MR secretome-encoding genes during the eutherian radiation are preferentially expressed in the epididymis. In contrast, only 42.9% (18/42) of the newly evolved secretome-encoding genes across the other six branches are epididymis-enriched. The direct contrast between enriched expression in testis versus epididymis confirmed that testis-enriched expression was disproportionately linked to genes originated in branches other than branch 5 (24/36), while epididymis-enriched expression was linked to genes originated in branch 5 (43/61; two-tailed Fishers exact test, 2TFET, *P* = 6.0 × 10^−4^) (Fig. 3A). This finding was reproduced when only genes with an ortholog expressed in the mouse epididymis (Trigg et al, 2026) were considered (19/25 vs 10/28; 2TFET, *P* = 5.4 × 10^−3^) (Appendix Table S4). Furthermore, this disparity became more pronounced when contrasting the enriched expression in the testis and epididymis of all genes in branch 5 of the MR secretome with that of the remaining branch 5 genes (43/50 vs 12/192; 2TFET, *P* = 6.2 × 10^−29^) (Fig. 3A). This result reinforces the notion that preferential expression in the epididymis is specific of male secreted protein-coding genes originated at branch 5 but not a general characteristic of all protein-encoding genes originated at such branch. The epididymis, a duct-like organ that enables the transit of the immature and nonmotile sperm generated by the testis to the *vas deferens*, is crucial for sperm to acquire motility and fertilization capability, as well as for sperm to be protected, temporally stored, and allowed to reach the right concentration (Bedford, 2014; Cuasnicu et al, 2002). Moreover, the epididymis contributes to sperm structural diversity and to a major molecular remodeling that involves lipid, epigenetic, and proteome changes associated with sperm maturation (Nixon et al, 2019a; Skerrett-Byrne et al, 2022; Teves and Roldan, 2022).

We used Gene Ontology (GO) terms to identify gene functions and determine enriched categories among the gained genes during the eutherian radiation. To minimize biases from relying on a single functional annotation source, we integrated results from three tools (DAVID, PANGEA, and PAN-GO) (“Methods”; Appendix Table S5). We compared enrichment patterns for the MR secretome genes associated with the radiation of eutheria (MR-*br5*) against those from the remaining MR secretome genes (MR-*rest*). Both gene sets share significant enrichment for GO terms related to secreted proteins (i.e., extracellular region), immunity, reproduction, and proteolysis (Fig. 2B). However, notable differences emerge in the specificity and magnitude of enrichment. For instance, MR-*rest* genes show stronger associations with terms related to reproduction and specifically fertilization (e.g., acrosomal vesicle), with some fertilization-specific terms being uniquely enriched in this group (e.g., sperm egg recognition). MR-*br5* genes show a more diverse array of Immune-related terms, with some being exclusive to genes in the eutherian branch (Fig. 3B). Finally, gene-enrichment associated with proteolysis is distinct between the two groups, with MR-*br5* showing significant enrichment for proteases that hydrolyze peptide bonds (peptidases), while MR-*rest* does with hydrolases and lysozymes (Fig. 3B). Counts of child terms found within these key parent categories show significant differences between number of terms unique to MR-*br5* or MR-*rest* (2TFET, *P* = 1.15 × 10^−10^) reflecting an abundance of reproduction terms uniquely associated with MR-*rest*, immunity genes with MR-*br5*, and an even count of proteolysis terms unique to each group (Fig. 3C; Appendix Table S5).

Beyond term-level enrichments, genes that originated during the eutherian radiation exemplify the functional innovations that characterized this evolutionary transition. Semenogelins (*SEMG1* and *SEMG2*), which are expressed in seminal vesicles, are known to form the semen coagulum, with *SEMG1* showing high rates of evolution leading to species with promiscuous mating systems (Kingan et al, 2003). Associations between bouts of positive selection and differences in mating types have been found for *SEMG2* in primates (Dorus et al, 2004; Ramm et al, 2008). Further, among the serine-endopeptidase-encoding genes found to originate during the eutherian radiation are *KLK1*, *KLK2*, and *KLK3*, which encode proteases that are part of the enzymatic pathway leading to semen liquefaction, a process that allows sperm to move freely within the female reproductive tract (Laflamme and Wolfner, 2013). The three *KLK* genes are prostate-enriched in expression. Recent enzyme kinetics analysis has revealed greater enzyme velocity and higher efficiency of the prostate-specific protease KLK3 in polygynandrous chimpanzees compared to other apes that experience lower levels of sperm competition (Kahveci et al, 2025). Notably, the MR-*br5* serine protease inhibitor Kunitz type 4 (*SPINT4*), which is epididymis-enriched in expression, has been shown to be essential for fertility in knockout experiments without detectable alteration of organ morphology or sperm parameters (Robertson et al, 2020).

Compared to MR-*rest*, MR-*br5* shows a broader immune-related profile (Fig. 3B), including a higher—although not statistically significant—proportion of β-defensins (22/72 vs. 8/51; 2TFET, *P* = 0.087). ß-defensins are known to be epididymis-enriched and have critical roles in sperm maturation and antimicrobial activity (Dorin and Barratt, 2014). An example is *DEFB126*, which, when mutated in human males, impairs sperm penetration of cervical mucus and reduces fertility without altering semen volume or sperm motility (Tollner et al, 2011). Likewise, epididymal protease inhibitor-encoding genes are present in both gene sets (six in MR-*br5* vs. four in MR-*rest*). These genes are central in sperm maturation and capacitation. Mice knockouts of some of the genes (*Wfdc9, Wfdc11*, and *Wfdc13*) originated during the eutherian radiation exhibit normal spermatogenesis and sperm counts but are infertile due to sperm motility defects and heightened sperm death (Kent et al, 2024). Further, protecting sperm from oxidative stress, two epididymis enriched in expression glutathione peroxidase-encoding genes, *GPX5* and *GPX6*, originated during the eutherian radiation (Pei et al, 2023). The knockout of *GPX5* in mice substantially affected the possibility of having healthy progeny of older relative to younger males (Chabory et al, 2009). Likewise, the epididymis-specific carboxyl esterase-encoding gene, *CES5A*, which is relevant in xenobiotic detoxification and lipid homeostasis, has been shown to be necessary for sperm capacitation and male fertility in rat knockouts (Ru et al, 2015).

Another interesting trend emerges when analyzing the presence of the MR secretome among the orthologous proteins in the mouse epididymis. An important secretory mode of protein and RNA delivery to sperm takes place within the epididymis via epididymosomes, which are key for sperm maturation and transgenerational paternal transmission of information (Chan et al, 2020; Nixon et al, 2019b). Among the nine genes with an epididymis-enriched expression profile in humans that have evidence of having an ortholog among 12 proteins found in mouse epididymosomes (Trigg et al, 2026), there are seven that originated at branch 5 (*CES5A, CLPSL2, GPX5, GPX6, LCN8, RNASE9*, and *SPINT4; CRISP1* and *RNASE12* originated in earlier branches). This finding suggests that the delivery of molecules by extracellular vesicles that influence pregnancy and offspring fitness was also impacted during the eutherian radiation.

In summary, the genes that contributed to the MR secretome and originated during the eutherian radiation show distinctive characteristics relative to genes that originated before or after this radiation (Fig. 3B,C). Genes in the latter set tend to encode constitutive proteins of the sperm. In contrast, genes that emerged during the eutherian radiation contribute to proteolytic pathways involved in postmating processes—such as sperm capacitation, protection against proteolysis, and seminal clot degradation—and mediate immune changes essential for sperm maturation and postmating protection. These key biological functions could contribute to the differential male reproductive success documented in many eutherians (Jones, 1998), which has been previously linked to changes in sperm and the ejaculate in the context of varying sperm competition levels (Lupold, 2013; Roldan et al, 1992). In fact, some of these genes have been proposed as potential male contraception targets for humans (Kent et al, 2024; Robertson et al, 2020).

The dramatic gene gain during the eutherian radiation affected the secretome produced by all male reproductive organs, but most markedly the epididymis. In eutherians, the epididymis is where complex sperm maturation occurs, including acrosomal matrix reorganization, sperm head rigidification, modifications of the sperm plasmalemma that mediate the initiation of sperm-zona binding, and shaping of the sperm epigenome, with the consequent potential alteration of paternal transgenerational effects (Bedford, 2014; Björkgren and Sipilä, 2019; Chan et al, 2020; Nixon et al, 2019b; Skerrett-Byrne et al, 2025). In addition to this sperm capacitation process, temporal storage of sperm in the cauda epididymis is thought to facilitate the production of recurrent sperm-rich ejaculates (Bedford, 2014). Our work provides a molecular foundation for understanding the drastic overhaul of the MR secretome during the eutherian radiation while highlighting the central role of the epididymis in this evolutionary innovation. Nevertheless, a detailed understanding of the evolutionary sequence of molecular changes underlying such an overhaul, as well as commonalities and more lineage-restricted changes in epididymis function, will only be possible once systematic transcriptomic and proteomic analyses of the epididymis are performed across the vertebrate phylogeny.

## Methods

**Table:** Reagents and tools table

Reagent/resource	Reference or source	Identifier or catalog number
**Experimental models**		
**Recombinant DNA**		
**Antibodies**		
**Oligonucleotides and other sequence-based reagents**		
**Chemicals, enzymes, and other reagents**		
**Software**		
DAVID	https://davidbioinformatics.nih.gov/	
DIOPT v9.0	https://www.flyrnai.org/cgi-bin/DRSC_orthologs_v9.pl	
PAN-GO Human Functionome	https://functionome.geneontology.org/	
PANGEA	https://www.flyrnai.org/tools/pangea/web/home/7227	
R	https://www.r-project.org/	
ShinyEpididymis	https://reproproteomics.shinyapps.io/ShinyEpididymis/	
The Human Protein Atlas v.24	https://www.proteinatlas.org/humanproteome/tissue/secretome	
**Other**		

### Human secretome data

Secretome-encoding genes were retrieved from the Human Protein Atlas v24.0 (https://www.proteinatlas.org/humanproteome/tissue/secretome) (Uhlen et al, 2019). Predicted secreted proteins were classified based on their primary site of secretion, taking into account the secreted protein tissue of origin. The secretomes delineated correspond to blood, brain, digestive system, female reproductive (FR) system, male reproductive (MR) system, immunoglobulins, intracellular and membrane (as a control), extracellular matrix, and other tissues. An additional category of unknown location was also retrieved. Gene expression profiles and expression-specificity across tissues were obtained from the same database (Uhlen et al, 2015; Uhlen et al, 2019).

### Phylogenetic gene age data

The human protein-coding genes considered and their phylogenetic mapping across the Bilateria phylogeny were obtained from Chen et al (Chen et al, 2025), which uses essentially the hg38_ver95 release, additional *Y-*linked genes, and an upgraded version of GenTree (http://gentree.ioz.ac.cn/) (Shao et al, 2019). With the exception of nine secretome-encoding genes, all the rest were present in Chen et al (Chen et al, 2025). Not present genes were associated with deprecated gene models in the most recent gene annotation releases. GenTree integrates genomic information from 23 species: human, chimp, orangutan, gibbon, rhesus monkey, marmoset, rabbit, guinea pig, rat, mouse, horse, dog, cow, elephant, opossum, platypus, chicken, zebra finch, lizard, western clawed frog, stickleback, pufferfish, and zebrafish.

### Calculation of the enrichment score

The enrichment score (ES_i,j_) quantifies the fold gain of new genes for a particular secretome (i) by branch (j) combination relative to the random expectation as:

$${{ES}}_{i,j}=\,\frac{{Proportion}\,{of}\,{gained}\,{genes}\,{by}\,{secretome}\,i\,{in}\,{branch}\,j}{{Overall}\,{proportion}\,{of}\,{gained}\,{genes}\,{in}\,{branch}\,j}=\,\frac{{n}_{i,j}/{N}_{i}}{{N}_{j}/T}$$

where *i* and *j* can be any combination of the ten secretomes and seven phylogenetic branches, respectively (see the main text). The variable *n*_*i,j*_ corresponds to the number of gained genes by secretome *i* in branch *j*, *N*_*i*_ to the total number of gained genes by the secretome *i, N*_*j*_ to the total number of gained genes by branch *j*, and *T* to the total number of genes considered across branches, i.e., 19,665. An ES value of 1 indicates no departure from random expectation, while values > 1 or <1 indicate that more or fewer genes than expected, respectively, were gained for a given secretome by branch combination. Statistical significance of ES values was assessed through Monte Carlo simulations (*n *= 100,000) using an in-house R script deposited in Zenodo (https://zenodo.org/records/18157698) (Ranz and Civetta, 2026). Simulations resampled genes without replacement under the assumption that genes are randomly distributed across secretomes and branches, with the constraint that each branch maintains a fixed number of genes (Chen et al, 2025; Shao et al, 2019). From these simulations, we calculated the 99% confidence interval (CI) and the mean simulated ES for each secretome by branch combination. Raw *P* values for the 70 ES values, one for each secretome by branch combination, were corrected for multiple testing (Benjamini and Hochberg, 1995). The ES metric implements normalization by branch size, defined as the fraction of genes originating at a given branch relative to the whole genome. Consequently, ES is more sensitive to deviations occurring in smaller branches compared to larger ones. In comparison, the χ² test of independence identifies the largest absolute departures between observed and expected gene counts for each secretome-branch combination relative to the proportion of genes in the entire genome originating at that same branch and secretome. Unlike the *χ*² test, ES accounts for differential branch sizes through its normalization approach.

### Gene data

Patterns of enrichment for GO terms across particular sets of human genes were explored with DAVID (Huang da et al, 2009), PANGEA (Hu et al, 2023), and the PAN-GO Human functionome (Feuermann et al, 2025). We analyzed the 72 MR secretome genes associated with the radiation of eutheria (MR-*br5*) using all human genes that originated during the eutherian radiation as background (Chen et al, 2025). The remaining 51 MR secretome genes (MR-*rest*) were analyzed using all human genes, except those that originated during the eutherian radiation, as background. For each group, we clustered redundant GO terms under parent terms using the rrvgo R package (Sayols, 2023). For each identified parental term, we calculated the median enrichment score (–log₁₀(*P*_adj_)) and the median fold enrichment (log_2_(FE)) of that term across the three tools. Paralogous relationships among secretome-encoding genes were established according to DIOPT v9.0 (Hu et al, 2011), with only high-confidence predictions being considered. Mouse epididymis proteomic data were obtained from the ShinyEpididymis database (https://reproproteomics.shinyapps.io/ShinyEpididymis/), which includes curated information from the epididymal tissue, epididymal sperm, and epididymosomes (Trigg et al, 2026).

### Statistical analyses

Test for equality of proportions, two-tailed Fisher’s exact test, *χ*^2^ test of independence, analysis of residuals, and the Benjamini–Hochberg correction for multiple testing were conducted using built-in functions in R (R Development Core Team, 2016). In the case of *χ*^2^ tests, the null hypothesis was tested by performing 2000 Monte Carlo simulations.

### Graphics

The synopsis image was created with BioRender.com (2026) (https://BioRender.com/e7fabmn).

## Supplementary information

Appendix

Peer Review File

Dataset EV1

Source data Fig. 1

Source data Fig. 2

Source data Fig. 3

## Data Availability

This study includes no data deposited in external repositories. The source data of this paper are collected in the following database record: biostudies:S-SCDT-10_1038-S44319-026-00817-1.
