# Supplementary material for: Male reproductive biology was reshaped during placental mammal diversification through epididymal secretome expansion
Source: EMBO Rep. 2026 Jun 3;27(13):3538–46. doi: 10.1038/s44319-026-00817-1 (PMC13354781; doi:10.1038/s44319-026-00817-1)
Supplement: Supplementary file 1 — Appendix [file 44319_2026_817_MOESM1_ESM.pdf]

Appendix For

Male reproductive biology was reshaped during placental mammal diversification through epididymal secretome expansion

Jose M. Ranz[1,2]* and Alberto Civetta[3]*

[1] Department of Ecology and Evolutionary Biology, University of California Irvine, CA 92697, USA

[2] Department of Systems Biology, University of California Irvine, CA 92697, USA

[3] Department of Biology, University of Winnipeg, Winnipeg, MB R3B 2E9, Canada

* To whom correspondence may be addressed:

Jose Ranz (jranz@uci.edu) and Alberto Civetta (a.civetta@uwinnipeg.ca)

Table of Contents:

Appendix Table S1, p. 4

Appendix Table S2, p. 5

Appendix Table S3, p. 6

Appendix Table S4, p. 7

Appendix Table S5, p. 8

Appendix References, p. 9

Paralogous relationships of the 72 MR secretome genes originated during the eutherian radiation

To better understand the context of origination of the 72 MR secretome genes during the eutherian radiation (Chen *et al*, 2025; Shao *et al*, 2019), we examined how many had a reliable paralog prediction. Using DIOPT v9.0 (Hu *et al*, 2011), and focusing only on high ranked predictions, we found that 36 (i.e., 50%) of these genes had a reliable paralog, forming 23 paralogroups (Appendix Table S3). Of those, five paralogroups, including two MR secretome-encoding genes each, showed no relationship with any other gene while the remaining 18 paralogroups, which included 26 MR secretome-encoding genes, also harbored the additional presence of 21 extras paralogous genes, meaning that they were absent in our initial list of 72 genes.

We explored more in detail how the branch of origin of the 26 MR secretome-encoding genes, and that of the 21 extra paralogous genes, are related as this could inform about daughter-parent relationships among these paralogs. Upon determining the branch of origin of the additional 21 genes, we found that eight originated prior to the eutherian radiation. These eight ancient paralogs were related to nine out of the 36 MR secretome-encoding genes, presumably being their parental copies. Notably, all those eight ancient paralogs were present in different secretomes, with three detected in the MR secretome. Together, these results suggest that functional specialization accompanied the expression evolution of at least six MR secretome paralogous genes that originated during the eutherian radiation.

Further, another 15 MR secretome-encoding genes that originated during the eutherian radiation featured paralogous relationships with nine other paralogs encoding secreted proteins and originated also during this same radiation. All these nine extra paralogs encode proteins that are present in secretomes other than the MR secretome. It is important to notice that two MR secretome-encoding genes originated during the eutherian radiation were related to both two older extra paralogs and one other extra paralog originated during the eutherian radiation. Lastly, four additional MR secretome-encoding genes originated during the eutherian radiation were found to have a paralogous relationship with four genes that map onto the younger branch represented by the primate radiation, all of them encoding MR secretome proteins.

Overall, with the information at hand, a scenario of duplication events involving genes originated prior to the eutherian radiation seems only plausible for seven of the MR secretome-encoding genes originated during such radiation. For the rest, a *de novo* origin cannot be discarded. Nevertheless, a more parsimonious explanation is that they resulted from duplication events

involving genes that no longer share enough sequence similarity to be recognized as paralogs, a possibility that warrants future detailed analyses.

Assessment of the effect of paralogous genes on the expansion of the MR secretome during the eutherian radiation

As a close inspection of the 72 genes of the MR secretome that originated during the eutherian radiation revealed an equal presence of single-copy genes and genes part of multigene families such as β-defensins, semenogelins, or the KLK serine-endopeptidases (Appendix Table S3), we interrogated the possibility that the excess of new genes in relation to the random expectation could be the result of a dramatic proliferation of multigene families during such radiation. First, we determined what secretome-encoding genes have at least one high-confident paralog prediction according to DIOPT v9.0 (Hu *et al.*, 2011) and repeated both the χ^2 test of independence and the Monte Carlo simulations with only those genes that did not have any high-confident prediction, *i.e.* potentially single-copy genes (Appendix Table S1; Fig. 2b). Eight-hundred and fifty-six genes fell into this last category. The MR secretome by branch 5 combination showed the second highest standardized residual (5.96) and the second highest enrichment score (5.18), both significant at 1%FDR (Benjamini & Hochberg, 1995). Although the intracellular membrane secretome by branch 0 combination featured the largest standardized residual (6.35), it did not exhibit a significant enrichment score at 1% FDR (1.03). Likewise, the immunoglobulin secretome by branch 3 combination exhibited the highest and statistically significant enrichment score at 1% FDR (6.12), but its standardized residual (3.16) was not statistically significant (P_{adj} = 0.087). From these results, it follows that the most solid gene gain by any measure, beyond multigene families, is that associated with the MR secretome during the eutherian radiation.

Appendix Table S1. Test for a nonrandom association between secretomes and branches of the vertebrate phylogeny

		Blood		Brain		Digestive		Extracellular Matrix		Female Reproductive	
**Analysis**	**Phylogenetic Branch ***	**Residual †**	P_{adj} *	**Residual †**	P_{adj} *	**Residual †**	P_{adj} *	**Residual**	P_{adj} *	**Residual †**	P_{adj} *
**All Genes**	***br0*, Euteleostomi & Older**	-4.24	$<1x10^{-6}$	4.29	$<1x10^{-6}$	-5.23	$<1x10^{-6}$	11.33	$<1x10^{-6}$	-2.59	0.08
	***br1*, Tetrapoda**	1.23	1	-0.125	1	0.95	1	-2.25	0.57	-0.88	1
	***br2*, Amniota**	2.82	0.17	0.46	1	-9.52	1	-1.54	1	-1	1
	***br3*, Mammalia**	0.7	1	-1.38	1	-1.05	1	-4.09	0.001	1.11	1
	***br4*, Theria**	6.03	$<1x10^{-6}$	-2.03	0.5	0.07	1	-2.14	0.45	3.3	0.02
	***br5*, Eutheria**	-2.23	0.3	-1.57	1	4.47	$<1x10^{-6}$	-6.13	$<1x10^{-6}$	-0.63	1
	***br6*, Primate**	0.08	1	-1.76	0.92	5.75	$<1x10^{-6}$	-3.2	0.032	4.76	$<1x10^{-6}$
**Only Genes Without High Confidence Paralog Predictions §**	***br0*, Euteleostomi & Older**	-4.41	$<1x10^{-6}$	1.86	0.55	-2.46	0.16	5	$<1x10^{-6}$	-1.42	1
	***br1*, Tetrapoda**	2.24	0.89	-0.36	1	0.85	1	-1.87	1	-0.14	1
	***br2*, Amniota**	2.35	1	0.17	1	-1.7	1	-0.31	1	-1.19	1
	***br3*, Mammalia**	0.4	1	-0.63	1	-1.16	1	-2.2	0.57	1.43	1
	***br4*, Theria**	2.75	0.21	-0.14	1	0.28	1	0.49	1	1.59	1
	***br5*, Eutheria**	-0.19	1	-0.49	1	3.07	0.037	-2.75	0.08	-0.65	1
	***br6*, Primate**	-0.82	1	-0.93	1	3.86	0.008	-1.5	1	2.71	0.24

		Immunoglobulin		Intracellular_Membrane		Male Reproductive		Other Tissues		Unknown Location	
**Analysis**	**Phylogenetic Branch ***	**Residual †**	P_{adj} *	**Residual †**	P_{adj} *	**Residual †**	P_{adj} *	**Residual †**	P_{adj} *	**Residual †**	P_{adj} *
**All Genes**	***br0*, Euteleostomi & Older**	-11	$<1x10^{-6}$	9.27	$<1x10^{-6}$	-8.85	$<1x10^{-6}$	-1.25	1	0.47	1
	***br1*, Tetrapoda**	3.93	0.003	0.17	1	-4.14	0.002	-0.14	1	1.09	1
	***br2*, Amniota**	-3.3	0.07	-1.65	1	-0.42	1	1.79	1	1.33	1
	***br3*, Mammalia**	7.89	$<1x10^{-6}$	-2.01	1	-0.39	1	1.69	1	-1.19	1
	***br4*, Theria**	-3.11	0.03	-3.71	0.007	1.81	0.7	-0.42	1	-0.84	1
	***br5*, Eutheria**	9.38	$<1x10^{-6}$	-6.24	$<1x10^{-6}$	14.54	$<1x10^{-6}$	-0.59	1	-0.56	1
	***br6*, Primate**	1.17	1	-3	0.04	3.07	0.04	0.39	1	-1.16	1
**Only Genes Without High Confidence Paralog Predictions §**	***br0*, Euteleostomi & Older**	-3.62	0.004	6.35	$<1x10^{-6}$	-3.83	0.002	-2.04	0.416	1.1	1
	***br1*, Tetrapoda**	0.12	1	-0.06	1	-2.26	0.878	-0.04	1	-0.5	1
	***br2*, Amniota**	-1.4	1	-1.35	1	0.31	1	0.59	1	-0.32	1
	***br3*, Mammalia**	3.16	0.087	-1.3	1	0.65	1	3.02	0.087	-2.14	0.571
	***br4*, Theria**	-1.41	1	-3.03	0.169	0.86	1	-0.18	1	-0.34	1
	***br5*, Eutheria**	3.82	0.003	-3.82	0.003	5.97	$<1x10^{-6}$	0.2	1	1.44	1
	***br6*, Primate**	2.14	0.76	-0.91	1	1	1	-0.5	1	-0.05	1

* Branch of origin within the vertebrate phylogeny (Chen et al, 2025): euteleostomi and older (br0), tetrapoda (br1), amniota (br2), mammalia (br3), theria (br4), eutheria (br5), and primate (br6).

† Negative and positive standardized residuals, and their corresponding p-values, are shown, denoting depletion and enrichment, respetively. When statistically siginificant, the adjusted p-value is shaded in red and green, respectively.

* After multiple test correction (Benjamini and Holberg, 1995).

§ According to DIOPT v9.0 (Hu et al, 2011).

Appendix Table S2. Test for a nonrandom association between sets of genes originated at particular phylogenetic branches and preferential tissue expression

	Branch 5 *		Remaining Branches *	
**Expression Enrichment Pattern**	**Residual †**	P_{adj} *	**Residual †**	P_{adj} *
**Testis**	-3.97	$<1x10^{-6}$	3.97	$<1x10^{-6}$
**Epididymis**	2.69	0.028	-2.69	0.028
**Prostate**	1.68	0.37	-1.68	0.37
**Seminal Vesicles**	1.45	0.59	-1.45	0.59

* Branch 5 refers to the eutherian radiation (Chen et al, 2025). The rest of the branches correspond to: euteleostomi and older (br0), tetrapoda (br1), amniota (br2), mammalia (br3), theria (br4), and primate (br6).

† Negative and positive standardized residuals, and their corresponding p-values, are shown, denoting depletion and enrichment, respetively. When statistically siginificant, the adjusted p-value is shaded in red and green, respectively.

‡ After multiple test correction (Benjamini and Holberg, 1995).

Appendix Table S3. Paralogous relationships of male reproductive secretome-encoding genes originated during the eutherian radiation

Gene	Ensembl ID *	Paralogroup ID §	Male Reproductive Secretome Branch 5 Presence? ‡	Gene Age †
BPIFA3	ENSG00000131059	1	1	br5
BPIFB1	ENSG00000125999	1	0	br3
CES1	ENSG00000198848	2	0	br1
CES5A	ENSG00000159398	2	1	br5
CST11	ENSG00000125831	3	1	br5
CST6	ENSG00000175315	3	0	br3
CST8	ENSG00000125815	3	1	br5
CST9	ENSG00000173335	4	0	br5
CST9L	ENSG00000101435	4	1	br5
DEFB104A	ENSG00000176782	5	0	br6
DEFB104B	ENSG00000177023	5	1	br5
DEFB105A	ENSG00000186562	6	1	br5
DEFB105B	ENSG00000186599	6	0	br6
DEFB106A	ENSG00000186579	7	0	br6
DEFB106B	ENSG00000187082	7	1	br5
DEFB115	ENSG00000215547	8	1	br5
DEFB118	ENSG00000131068	8	1	br5
DEFB119	ENSG00000180483	8	1	br5
DEFB121	ENSG00000204548	8	1	br5
DEFB123	ENSG00000180424	8	0	br5
DEFB125	ENSG00000178591	8	1	br5
DEFB135	ENSG00000205883	8	1	br5
DEFB127	ENSG00000088782	9	1	br5
DEFB128	ENSG00000185982	9	1	br5
DKK3	ENSG00000050165	10	0	br0
DKKL1	ENSG00000104901	10	1	br5
EDDM3A	ENSG00000181562	11	0	br4
EDDM3B	ENSG00000181552	11	1	br5
KLK1	ENSG00000167748	12	0	br5
KLK2	ENSG00000167751	12	1	br5
KLK3	ENSG00000142515	12	1	br5
LCN2	ENSG00000148346	13	0	br5
LCN9	ENSG00000148386	13	1	br5
LY6K	ENSG00000160886	14	1	br5
GML	ENSG00000104499	14	0	br5
OOSP2	ENSG00000149507	15	1	br5
PLAC1	ENSG00000170965	15	0	br3
PRSS38	ENSG00000185888	16	1	br5
PRSS48	ENSG00000189099	16	0	br5
RNASE10	ENSG00000182545	17	0	br5
RNASE11	ENSG00000173464	17	1	br5
RNASE12	ENSG00000258436	17	0	br4
RNASE13	ENSG00000206150	17	0	br3
RNASE9	ENSG00000188655	17	1	br5
SEMG1	ENSG00000124233	18	1	br5
SEMG2	ENSG00000124157	18	1	br5
SPAG11A	ENSG00000178287	19	0	br6
SPAG11B	ENSG00000164871	19	1	br5
LYPD4	ENSG00000273111	20	0	br5
TEX101	ENSG00000131126	20	1	br5
CD177	ENSG00000204936	20	0	br5
WFDC10A	ENSG00000180305	21	1	br5
WFDC10B	ENSG00000182931	21	1	br5
WFDC11	ENSG00000180083	22	1	br5
WFDC9	ENSG00000180205	22	1	br5
WFDC6	ENSG00000243543	23	1	br5
SPINT4	ENSG00000149651	23	1	br5

* From the hg38_ver95 release.

§ According to DIOPT v9.0 (Hu et al, 2011). High rank predictions only.

‡ 1 and 0, present or nonpresent among the 72 male reproductive secretome-encoding genes originated during the eutherian radiation (i.e. branch 5).

† Branch of origin within the vertebrate phylogeny (Chen et al, 2025): euteleostomi and older (br0), tetrapoda (br1), amniota (br2), mammalia (br3), theria (br4), eutheria (br5), and primate (br6).

Appendix Table S4. Human tissue-enriched genes in expression with detectable protein expression of their orthologs across different compartments of the mouse epididimys

Human Genes				Mouse Epididymis ‡			
**ENSG**	**Name**	**Gene Age ***	**T_E_P_S †**	**Sperm**	**Epididymosomes**	**Tissue**	**Found**
ENSG00000158525	CPA5	0	1_0_0_0	1	0	0	1
ENSG00000124490	CRISP2	0	1_0_0_0	1	0	0	1
ENSG00000139549	DHH	0	1_0_0_0	1	1	1	1
ENSG00000100312	ACR	2	1_0_0_0	1	1	1	1
ENSG00000111644	ACRBP	2	1_0_0_0	1	0	1	1
ENSG00000173401	GLIPR1L1	2	1_0_0_0	1	0	1	1
ENSG00000103023	PRSS54	2	1_0_0_0	1	0	0	1
ENSG00000042813	ZPBP	2	1_0_0_0	1	1	1	1
ENSG00000186075	ZPBP2	2	1_0_0_0	1	0	0	1
ENSG00000124812	CRISP1	3	0_1_0_0	1	1	1	1
ENSG00000206150	RNASE13	3	0_1_0_0	1	0	1	1
ENSG00000099338	CATSPERG	3	1_0_0_0	1	0	0	1
ENSG00000120563	LYZL1	3	1_0_0_0	1	0	0	1
ENSG00000157093	LYZL4	3	1_0_0_0	1	0	0	1
ENSG00000275722	LYZL6	3	1_0_0_0	1	0	0	1
ENSG00000258484	SPESP1	3	1_0_0_0	1	0	0	1
ENSG00000184925	LCN12	4	0_1_0_0	1	0	0	1
ENSG00000258436	RNASE12	4	0_1_0_0	1	1	1	1
ENSG00000158901	WFDC8	4	0_1_0_0	1	0	1	1
ENSG00000120160	EQTN	4	1_0_0_0	1	0	0	1
ENSG00000165076	PRSS37	4	1_0_0_0	1	0	0	1
ENSG00000141316	SPACA3	4	1_0_0_0	1	0	0	1
ENSG00000153498	SPACA7	4	1_0_0_0	1	0	0	1
ENSG00000188334	BSPH1	5	0_1_0_0	1	0	0	1
ENSG00000159398	CES5A	5	0_1_0_0	1	1	1	1
ENSG00000196748	CLPSL2	5	0_1_0_0	1	1	1	1
ENSG00000125831	CST11	5	0_1_0_0	1	0	1	1
ENSG00000214642	DEFB113	5	0_1_0_0	0	0	1	1
ENSG00000267710	EDDM13	5	0_1_0_0	1	0	0	1
ENSG00000181552	EDDM3B	5	0_1_0_0	1	0	0	1
ENSG00000224586	GPX5	5	0_1_0_0	1	1	1	1
ENSG00000198704	GPX6	5	0_1_0_0	1	1	1	1
ENSG00000267206	LCN6	5	0_1_0_0	1	0	0	1
ENSG00000204001	LCN8	5	0_1_0_0	1	1	1	1
ENSG00000204583	LRCOL1	5	0_1_0_0	1	0	0	1
ENSG00000171053	PATE1	5	0_1_0_0	1	0	0	1
ENSG00000173464	RNASE11	5	0_1_0_0	1	0	0	1
ENSG00000188655	RNASE9	5	0_1_0_0	1	1	1	1
ENSG00000164871	SPAG11B	5	0_1_0_0	1	0	0	1
ENSG00000214510	SPINK13	5	0_1_0_0	1	0	0	1
ENSG00000149651	SPINT4	5	0_1_0_0	1	1	0	1
ENSG00000168634	WFDC13	5	0_1_0_0	1	0	0	1
ENSG00000134940	ACRV1	5	1_0_0_0	1	0	1	1
ENSG00000131059	BPIFA3	5	1_0_0_0	1	0	0	1
ENSG00000125815	CST8	5	1_0_0_0	1	0	0	1
ENSG00000125823	CSTL1	5	1_0_0_0	1	0	0	1
ENSG00000104901	DKKL1	5	1_0_0_0	1	0	0	1
ENSG00000203795	FAM24A	5	1_0_0_0	1	0	0	1
ENSG00000099840	IZUMO4	5	1_0_0_0	1	0	0	1
ENSG00000185888	PRSS38	5	1_0_0_0	1	0	0	1
ENSG00000171489	SPACA5	5	1_0_0_0	1	0	0	1
ENSG00000131126	TEX101	5	1_0_0_0	1	1	1	1
ENSG00000178287	SPAG11A	6	0_1_0_0	1	0	1	1
ENSG00000188992	LIPI	1	0_1_0_0	0	0	0	0
ENSG00000166845	C18orf54	1	1_0_0_0	0	0	0	0
ENSG00000104804	TULP2	2	1_0_0_0	0	0	0	0
ENSG00000169393	ELSPBP1	3	0_1_0_0	0	0	0	0
ENSG00000203970	DEFB110	4	0_1_0_0	0	0	0	0
ENSG00000181562	EDDM3A	4	0_1_0_0	0	0	0	0
ENSG00000196844	PATE2	4	0_1_0_0	0	0	0	0
ENSG00000165131	LLCFC1	4	1_0_0_0	0	0	0	0
ENSG00000125900	SIRPD	4	1_0_0_0	0	0	0	0
ENSG00000243543	WFDC6	5	0_1_0_0	0	0	0	0
ENSG00000237353	PATE4	5	0_0_0_1	0	0	0	0
ENSG00000124233	SEMG1	5	0_0_0_1	0	0	0	0
ENSG00000124157	SEMG2	5	0_0_0_1	0	0	0	0
ENSG00000167751	KLK2	5	0_0_1_0	0	0	0	0
ENSG00000142515	KLK3	5	0_0_1_0	0	0	0	0
ENSG00000167749	KLK4	5	0_0_1_0	0	0	0	0
ENSG00000263639	MSMB	5	0_0_1_0	0	0	0	0
ENSG00000186562	DEFB105A	5	0_1_0_0	0	0	0	0
ENSG00000187082	DEFB106B	5	0_1_0_0	0	0	0	0
ENSG00000198129	DEFB107B	5	0_1_0_0	0	0	0	0
ENSG00000206034	DEFB109B	5	0_1_0_0	0	0	0	0
ENSG00000177684	DEFB114	5	0_1_0_0	0	0	0	0
ENSG00000215547	DEFB115	5	0_1_0_0	0	0	0	0
ENSG00000131068	DEFB118	5	0_1_0_0	0	0	0	0
ENSG00000180483	DEFB119	5	0_1_0_0	0	0	0	0
ENSG00000204548	DEFB121	5	0_1_0_0	0	0	0	0
ENSG00000125788	DEFB126	5	0_1_0_0	0	0	0	0
ENSG00000088782	DEFB127	5	0_1_0_0	0	0	0	0
ENSG00000185982	DEFB128	5	0_1_0_0	0	0	0	0
ENSG00000125903	DEFB129	5	0_1_0_0	0	0	0	0
ENSG00000233050	DEFB130B	5	0_1_0_0	0	0	0	0
ENSG00000186458	DEFB132	5	0_1_0_0	0	0	0	0
ENSG00000205882	DEFB134	5	0_1_0_0	0	0	0	0
ENSG00000205883	DEFB135	5	0_1_0_0	0	0	0	0
ENSG00000205884	DEFB136	5	0_1_0_0	0	0	0	0
ENSG00000139574	NPFF	5	0_1_0_0	0	0	0	0
ENSG00000236027	PATE3	5	0_1_0_0	0	0	0	0
ENSG00000180305	WFDC10A	5	0_1_0_0	0	0	0	0
ENSG00000180083	WFDC11	5	0_1_0_0	0	0	0	0
ENSG00000180205	WFDC9	5	0_1_0_0	0	0	0	0
ENSG00000149507	OOSP2	5	1_0_0_0	0	0	0	0
ENSG00000258223	PRSS58	5	1_0_0_0	0	0	0	0
ENSG00000176782	DEFB104A	6	0_1_0_0	0	0	0	0
ENSG00000186599	DEFB105B	6	0_1_0_0	0	0	0	0
ENSG00000186579	DEFB106A	6	0_1_0_0	0	0	0	0
ENSG00000186572	DEFB107A	6	0_1_0_0	0	0	0	0
ENSG00000184276	DEFB108B	6	0_1_0_0	0	0	0	0
ENSG00000232948	DEFB130A	6	0_1_0_0	0	0	0	0
ENSG00000225805	DEFB131B	6	0_1_0_0	0	0	0	0
ENSG00000151033	LYZL2	6	1_0_0_0	0	0	0	0
ENSG00000171478	SPACA5B	6	1_0_0_0	0	0	0	0

* Branch of origin within the vertebrate phylogeny (Chen et al, 2025): euteleostomi and older (br0), tetrapoda (br1), amniota (br2), mammalia (br3), theria (br4), eutheria (br5), and primate (br6).
† 1, preferential expression; 0, no preferential expression. T= Testis; E= Epididymis; P= Prostate; S= Seminal Vesicle.
‡ According to ShinyEpididymis (https://reproproteomics.shinyapps.io/ShinyEpididymis/).

Appendix Table S5. Outcome of the functional enrichment analysis for male reproductive secretome genes originated during the eutherian radiaton and for those in the rest of the vertebrate phylogeny

MR-*br5* Gene Set — Enrichment Patterns

Tool	GO Rubric *	GO.ID	ParentTerm	Term	*P-Value*	FE §	Log2(FE)	Padj ¶	Score = Log10(Padj)
DAVID	BP	GO:0002376	antimicrobial humoral response	immune system process	1.17E-04	1.954	0.966244	0.002116122	2.674
DAVID	BP	GO:0006955	antimicrobial humoral response	immune response	2.09E-05	2.136	1.094759	4.92E-04	3.308
DAVID	BP	GO:0006959	antimicrobial humoral response	humoral immune response	1.20E-04	3.878	1.955356	0.002116122	2.674
DAVID	BP	GO:0019730	antimicrobial humoral response	antimicrobial humoral response	9.90E-06	4.996	2.320641	2.50E-04	3.603
DAVID	BP	GO:0019731	antimicrobial humoral response	antibacterial humoral response	8.81E-04	5.731	2.518785	0.012962448	1.887
DAVID	BP	GO:0031640	biological process involved in interspecies	killing of cells of another organism	0.002398366	4.776	2.255751	0.031356416	1.504
DAVID	BP	GO:0044419	biological process involved in interspecies	biological process involved in interspecies interac	6.28E-14	3.745	1.904884	3.17E-12	11.499
DAVID	BP	GO:0006950	defense response	response to stress	7.95E-12	2.987	1.578793	2.55E-10	9.593
DAVID	BP	GO:0006952	defense response	defense response	1.44E-13	3.647	1.86658	6.36E-12	11.197
DAVID	BP	GO:0050896	defense response	response to stimulus	6.64E-04	1.445	0.530858	0.010192346	1.992
DAVID	BP	GO:0009607	defense response to bacterium	response to biotic stimulus	2.66E-14	3.848	1.944233	1.56E-12	11.806
DAVID	BP	GO:0009617	defense response to bacterium	response to bacterium	5.27E-22	6.869	2.780072	9.30E-20	19.032
DAVID	BP	GO:0042742	defense response to bacterium	defense response to bacterium	2.18E-25	8.532	3.09286	7.71E-23	22.113
DAVID	BP	GO:0043207	defense response to bacterium	response to external biotic stimulus	2.30E-14	3.866	1.950896	1.56E-12	11.806
DAVID	BP	GO:0045087	defense response to bacterium	innate immune response	8.23E-09	3.791	1.922715	2.24E-07	6.651
DAVID	BP	GO:0050829	defense response to bacterium	defense response to Gram-negative bacterium	6.15E-12	10.526	3.395929	2.17E-10	9.663
DAVID	BP	GO:0050830	defense response to bacterium	defense response to Gram-positive bacterium	1.62E-04	5.263	2.395929	0.002715284	2.566
DAVID	BP	GO:0051707	defense response to bacterium	response to other organism	2.30E-14	3.866	1.950896	1.56E-12	11.806
DAVID	BP	GO:0098542	defense response to bacterium	defense response to other organism	3.20E-15	4.114	2.040448	3.77E-13	12.424
DAVID	BP	GO:0140546	defense response to bacterium	defense response to symbiont	5.13E-11	3.779	1.917881	1.51E-09	8.822
DAVID	BP	GO:0009605	response to external stimulus	response to external stimulus	2.47E-13	3.584	1.841598	9.69E-12	11.014
DAVID	BP	GO:0050918	response to external stimulus	positive chemotaxis	0.00294561	12.281	3.618321	0.037135721	1.430
DAVID	BP	GO:0007338	sexual reproduction	single fertilization	4.01E-05	9.552	3.255751	8.32E-04	3.080
DAVID	BP	GO:0009566	sexual reproduction	fertilization	1.07E-04	8.187	3.033359	0.002091042	2.680
DAVID	BP	GO:0019953	sexual reproduction	sexual reproduction	2.93E-05	4.094	2.033359	6.47E-04	3.189
DAVID	BP	GO:0022414	sexual reproduction	reproductive process	3.97E-04	3.161	1.660549	0.006367149	2.196
DAVID	BP	GO:0048609	sexual reproduction	multicellular organismal reproductive process	0.003700536	3.349	1.743852	0.045044457	1.346
DAVID	CC	GO:0001669	acrosomal vesicle	acrosomal vesicle	3.48E-04	8.711	3.122857	0.010454548	1.981
DAVID	CC	GO:0005576	extracellular region	extracellular region	3.07E-37	3.283	1.714949	1.84E-35	34.735
DAVID	MF	GO:0001530	lipopolysaccharide binding	lipopolysaccharide binding	4.69E-04	11.833	3.564785	0.002673166	2.573
DAVID	MF	GO:0008289	lipopolysaccharide binding	lipid binding	0.003891723	4.360	2.124212	0.018485682	1.733
DAVID	MF	GO:0004857	peptidase inhibitor activity	enzyme inhibitor activity	8.49E-08	6.837	2.773371	6.91E-07	6.160
DAVID	MF	GO:0004866	peptidase inhibitor activity	endopeptidase inhibitor activity	6.49E-10	9.925	3.311028	1.85E-08	7.733
DAVID	MF	GO:0004867	peptidase inhibitor activity	serine-type endopeptidase inhibitor activity	5.47E-08	13.312	3.73471	5.19E-07	6.285
DAVID	MF	GO:0030234	peptidase inhibitor activity	enzyme regulator activity	1.89E-07	4.855	2.279382	1.20E-06	5.922
DAVID	MF	GO:0030414	peptidase inhibitor activity	peptidase inhibitor activity	6.49E-10	9.925	3.311028	1.85E-08	7.733
DAVID	MF	GO:0042056	peptidase inhibitor activity	chemoattractant activity	0.002118975	13.524	3.75743	0.010980145	1.959
DAVID	MF	GO:0061134	peptidase inhibitor activity	peptidase regulator activity	9.98E-09	8.096	3.017297	1.42E-07	6.847
DAVID	MF	GO:0061135	peptidase inhibitor activity	endopeptidase regulator activity	4.92E-09	8.546	3.095299	9.34E-08	7.030
DAVID	MF	GO:0098772	peptidase inhibitor activity	molecular function regulator activity	1.41E-08	3.550	1.827819	1.61E-07	6.792
PANGEA	BP	GO:0009988	cell-cell recognition	cell-cell recognition	0.002268392	10.101	3.336	0.028200875	1.550
PANGEA	BP	GO:0006952	defense response	defense response	4.15E-11	3.518	1.815	3.21E-09	8.493
PANGEA	BP	GO:0061760	defense response	antifungal innate immune response	0.001336861	11.785	3.559	0.018697492	1.728
PANGEA	BP	GO:0009617	defense response to bacterium	response to bacterium	2.57E-18	6.510	2.703	3.70E-16	15.431
PANGEA	BP	GO:0042742	defense response to bacterium	defense response to bacterium	1.69E-22	8.763	3.131	3.41E-20	19.467
PANGEA	BP	GO:0043207	defense response to bacterium	response to external biotic stimulus	7.12E-13	3.928	1.974	7.17E-11	10.144
PANGEA	BP	GO:0045087	defense response to bacterium	innate immune response	1.02E-11	4.533	2.18	8.58E-10	9.066
PANGEA	BP	GO:0050829	defense response to bacterium	defense response to Gram-negative bacterium	3.34E-10	11.272	3.495	2.10E-08	7.677
PANGEA	BP	GO:0050830	defense response to bacterium	defense response to Gram-positive bacterium	8.28923E-05	7.443	2.896	0.001490582	2.827
PANGEA	BP	GO:0050918	defense response to bacterium	positive chemotaxis	0.003519289	8.839	3.144	0.041693225	1.380
PANGEA	BP	GO:0051707	defense response to bacterium	response to other organism	7.12E-13	3.928	1.974	7.17E-11	10.144
PANGEA	BP	GO:0098542	defense response to bacterium	defense response to other organism	1.45E-13	4.312	2.108	1.83E-11	10.738
PANGEA	BP	GO:0002376	immune response	immune system process	3.93E-07	2.388	1.256	1.31942E-05	4.880
PANGEA	BP	GO:0006955	immune response	immune response	3.42E-08	2.638	1.399	1.4368E-06	5.843
PANGEA	BP	GO:0006959	immune response	humoral immune response	3.80391E-05	4.533	2.18	0.000751086	3.124
PANGEA	BP	GO:0019730	immune response	antimicrobial humoral response	1.06E-07	8.127	3.023	4.09759E-06	5.387
PANGEA	BP	GO:0019731	immune response	antibacterial humoral response	1.85208E-06	12.856	3.684	4.54888E-05	4.342
PANGEA	BP	GO:0006508	negative regulation of endopeptidase acti	proteolysis	0.000182725	3.064	1.615	0.00318705	2.497
PANGEA	BP	GO:0010466	negative regulation of endopeptidase acti	negative regulation of peptidase activity	3.11E-08	9.065	3.18	1.36078E-06	5.866
PANGEA	BP	GO:0010951	negative regulation of endopeptidase acti	negative regulation of endopeptidase activity	3.11E-08	9.065	3.18	1.36078E-06	5.866
PANGEA	BP	GO:0030162	negative regulation of endopeptidase acti	regulation of proteolysis	1.47781E-05	5.015	2.326	0.000310032	3.509
PANGEA	BP	GO:0045861	negative regulation of endopeptidase acti	negative regulation of proteolysis	1.53E-07	7.857	2.974	5.7201E-06	5.243
PANGEA	BP	GO:0051248	negative regulation of endopeptidase acti	negative regulation of protein metabolic process	3.68951E-06	5.185	2.374	8.64032E-05	4.063
PANGEA	BP	GO:0052547	negative regulation of endopeptidase acti	regulation of peptidase activity	1.83595E-06	6.203	2.633	4.54888E-05	4.342
PANGEA	BP	GO:0052548	negative regulation of endopeptidase acti	regulation of endopeptidase activity	1.83595E-06	6.203	2.633	4.54888E-05	4.342
PANGEA	BP	GO:0010469	negative regulation of hydrolase activity	regulation of signaling receptor activity	0.002268392	10.101	3.336	0.028200875	1.550
PANGEA	BP	GO:0043086	negative regulation of hydrolase activity	negative regulation of catalytic activity	1.18883E-06	5.761	2.526	3.23556E-05	4.490
PANGEA	BP	GO:0050790	negative regulation of hydrolase activity	regulation of catalytic activity	3.13452E-05	3.041	1.605	0.000631293	3.200
PANGEA	BP	GO:0051346	negative regulation of hydrolase activity	negative regulation of hydrolase activity	7.17E-08	8.418	3.073	2.88895E-06	5.539
PANGEA	BP	GO:0032094	response to food	response to food	0.001336861	11.785	3.559	0.018697492	1.728
PANGEA	BP	GO:0050896	response to stimulus	response to stimulus	8.27291E-05	1.589	0.668	0.001490582	2.827
PANGEA	BP	GO:0007338	single fertilization	single fertilization	6.1978E-06	8.684	3.118	0.000141845	3.848
PANGEA	BP	GO:0009566	single fertilization	fertilization	1.89312E-05	7.499	2.907	0.000389055	3.410
PANGEA	BP	GO:0019953	single fertilization	sexual reproduction	0.002461271	3.251	1.701	0.030225615	1.520
PANGEA	CC	GO:0001669	acrosomal vesicle	acrosomal vesicle	4.53431E-05	10.713	3.421	0.000878087	3.056
PANGEA	CC	GO:0005576	extracellular region	extracellular region	1.00E-25	3.867	1.951	3.36E-23	22.474
PANGEA	MF	GO:0003824	catalytic activity	catalytic activity	0.000458479	1.951	0.964	0.007568659	2.121
PANGEA	MF	GO:0042379	CCR chemokine receptor binding	chemokine receptor binding	0.001790684	5.357	2.421	0.024042913	1.619
PANGEA	MF	GO:0048020	CCR chemokine receptor binding	CCR chemokine receptor binding	0.000497011	6.932	2.793	0.008072417	2.093
PANGEA	MF	GO:0004175	endopeptidase activity	endopeptidase activity	2.15E-07	5.472	2.452	7.74263E-06	5.111
PANGEA	MF	GO:0004252	endopeptidase activity	serine-type endopeptidase activity	7.90E-07	6.734	2.751	2.33971E-05	4.631
PANGEA	MF	GO:0008233	endopeptidase activity	peptidase activity	1.38183E-06	4.714	2.237	3.66185E-05	4.436
PANGEA	MF	GO:0008236	endopeptidase activity	serine-type peptidase activity	1.05705E-06	6.547	2.711	2.95681E-05	4.529
PANGEA	MF	GO:0016787	endopeptidase activity	hydrolase activity	6.086E-05	2.763	1.466	0.001156341	2.937
PANGEA	MF	GO:0017171	endopeptidase activity	serine hydrolase activity	1.05705E-06	6.547	2.711	2.95681E-05	4.529
PANGEA	MF	GO:0001530	lipopolysaccharide binding	lipopolysaccharide binding	0.000689423	14.142	3.822	0.010518919	1.978
PANGEA	MF	GO:0008289	lipopolysaccharide binding	lipid binding	0.003348344	3.928	1.974	0.040623883	1.391
PANGEA	MF	GO:0004857	peptidase inhibitor activity	enzyme inhibitor activity	5.84E-07	6.932	2.793	1.78173E-05	4.749
PANGEA	MF	GO:0004866	peptidase inhibitor activity	endopeptidase inhibitor activity	1.23E-08	9.821	3.296	6.19E-07	6.209
PANGEA	MF	GO:0030234	peptidase inhibitor activity	enzyme regulator activity	4.17E-07	5.193	2.377	1.34207E-05	4.872
PANGEA	MF	GO:0030414	peptidase inhibitor activity	peptidase inhibitor activity	1.23E-08	9.821	3.296	6.19E-07	6.209
PANGEA	MF	GO:0030545	peptidase inhibitor activity	signaling receptor regulator activity	0.001532361	3.492	1.804	0.021138184	1.675
PANGEA	MF	GO:0042056	peptidase inhibitor activity	chemoattractant activity	0.002268392	10.101	3.336	0.028200875	1.550
PANGEA	MF	GO:0061135	peptidase inhibitor activity	endopeptidase regulator activity	3.11E-08	9.065	3.18	1.36078E-06	5.866
PANGEA	MF	GO:0002020	protease binding	protease binding	4.26E-07	7.142	2.836	1.34207E-05	4.872
PAN-GO	BP	GO:0021700	anatomical structure maturation	developmental maturation	3.47E-04	16.530	4.047015	2.81E-02	1.551
PAN-GO	BP	GO:0048240	anatomical structure maturation	sperm capacitation	3.47E-04	16.530	4.047015	2.72E-02	1.565
PAN-GO	BP	GO:0048469	anatomical structure maturation	cell maturation	3.47E-04	16.530	4.047015	2.63E-02	1.580
PAN-GO	BP	GO:0071695	anatomical structure maturation	anatomical structure maturation	3.47E-04	16.530	4.047015	2.54E-02	1.595
PAN-GO	BP	GO:0009607	defense response to bacterium	response to biotic stimulus	7.10E-04	2.680	1.422233	4.35E-02	1.362
PAN-GO	BP	GO:0009617	defense response to bacterium	response to bacterium	2.29E-05	4.330	2.114367	2.16E-03	2.666
PAN-GO	BP	GO:0019730	defense response to bacterium	antimicrobial humoral response	9.70E-06	8.120	3.02148	1.05E-03	2.979
PAN-GO	BP	GO:0019731	defense response to bacterium	antibacterial humoral response	8.47E-06	13.780	3.784504	9.60E-04	3.018
PAN-GO	BP	GO:0042742	defense response to bacterium	defense response to bacterium	1.83E-06	5.510	2.462052	2.18E-04	3.662
PAN-GO	BP	GO:0043207	defense response to bacterium	response to external biotic stimulus	6.47E-04	2.700	1.432959	4.19E-02	1.378
PAN-GO	BP	GO:0051707	defense response to bacterium	response to other organism	6.47E-04	2.700	1.432959	4.08E-02	1.389
PAN-GO	BP	GO:0098542	defense response to bacterium	defense response to other organism	2.64E-04	2.950	1.560715	2.22E-02	1.654
PAN-GO	BP	GO:0006952	response to external stimulus	defense response	5.89E-04	2.730	1.448901	3.93E-02	1.406
PAN-GO	BP	GO:0009605	response to external stimulus	response to external stimulus	2.79E-05	3.070	1.618239	2.53E-03	2.597
PAN-GO	BP	GO:0032094	response to food	response to food	8.98E-05	22.040	4.462052	7.83E-03	2.106
PAN-GO	BP	GO:0019953	sexual reproduction	sexual reproduction	4.96E-04	6.890	2.784504	3.41E-02	1.467
PAN-GO	MF	GO:0016209	antioxidant activity	antioxidant activity	1.23E-04	12.590	3.654206	4.44E-03	2.353
PAN-GO	MF	GO:0004601	glutathione peroxidase activity	peroxidase activity	1.23E-04	12.590	3.654206	4.67E-03	2.331
PAN-GO	MF	GO:0004602	glutathione peroxidase activity	glutathione peroxidase activity	8.98E-05	22.040	4.462052	3.59E-03	2.445
PAN-GO	MF	GO:0016684	glutathione peroxidase activity	oxidoreductase activity, acting on peroxide as acc	1.23E-04	12.590	3.654206	4.23E-03	2.374
PAN-GO	MF	GO:0004857	serine-type endopeptidase inhibitor activit	enzyme inhibitor activity	9.06E-04	6.120	2.613532	2.33E-02	1.633
PAN-GO	MF	GO:0004866	serine-type endopeptidase inhibitor activit	endopeptidase inhibitor activity	1.63E-04	8.480	3.084064	4.68E-03	2.330
PAN-GO	MF	GO:0004867	serine-type endopeptidase inhibitor activit	serine-type endopeptidase inhibitor activity	8.47E-06	13.780	3.784504	3.81E-04	3.419
PAN-GO	MF	GO:0030414	serine-type endopeptidase inhibitor activit	peptidase inhibitor activity	1.63E-04	8.480	3.084064	4.50E-03	2.347
PAN-GO	MF	GO:0048019	serine-type endopeptidase inhibitor activit	receptor antagonist activity	2.03E-03	22.040	4.462052	4.87E-02	1.312
PAN-GO	MF	GO:0061134	serine-type endopeptidase inhibitor activit	peptidase regulator activity	2.44E-04	7.870	2.976364	6.50E-03	2.187
PAN-GO	MF	GO:0061135	serine-type endopeptidase inhibitor activit	endopeptidase regulator activity	1.63E-04	8.480	3.084064	4.87E-03	2.312
PAN-GO	MF	GO:0098772	serine-type endopeptidase inhibitor activit	molecular function regulator activity	1.37E-03	2.980	1.575312	3.40E-02	1.469

MR-*rest* Gene Set — Enrichment Patterns

Tool	GO Rubric *	GO.ID	ParentTerm	Term	*P*-Value	FE §	Log2(FE)	P_{adj} ¶	Score = Log10(P_{adj})
DAVID	BP	GO:004441	biological process involved in inters	biological process involved in interspecies interacti	2.32102E-05	3.869	1.951824	0.00032797	3.484
DAVID	BP	GO:000695	defense response	defense response	9.47323E-06	4.204	2.07172	0.000139945	3.854
DAVID	BP	GO:000960	defense response	response to external stimulus	0.000234075	3.095	1.6301	0.003042981	2.517
DAVID	BP	GO:000960	defense response to bacterium	response to biotic stimulus	9.08511E-06	4.220	2.077262	0.000139945	3.854
DAVID	BP	GO:000961	defense response to bacterium	response to bacterium	1.36231E-09	9.016	3.172521	4.42751E-08	7.354
DAVID	BP	GO:004274	defense response to bacterium	defense response to bacterium	1.40738E-14	22.467	4.489711	7.62329E-13	12.118
DAVID	BP	GO:004320	defense response to bacterium	response to external biotic stimulus	6.51087E-06	4.351	2.12124	0.000105802	3.976
DAVID	BP	GO:004508	defense response to bacterium	innate immune response	0.001228708	4.655	2.21863	0.013311	1.876
DAVID	BP	GO:005083	defense response to bacterium	defense response to Gram-positive bacterium	0.001796544	16.147	4.01318	0.018246153	1.739
DAVID	BP	GO:005170	defense response to bacterium	response to other organism	6.34286E-06	4.361	2.124679	0.000105802	3.976
DAVID	BP	GO:009854	defense response to bacterium	defense response to other organism	1.46792E-07	6.084	2.605065	2.98172E-06	5.526
DAVID	BP	GO:014054	defense response to bacterium	defense response to symbiont	7.33376E-05	5.235	2.388127	0.000993113	3.003
DAVID	BP	GO:000167	fertilization	acrosome assembly	0.003614615	32.630	5.028131	0.033564281	1.474
DAVID	BP	GO:000727	fertilization	gamete generation	0.000681669	4.405	2.139162	0.008205281	2.086
DAVID	BP	GO:000728	fertilization	spermatogenesis	0.000789666	5.020	2.327691	0.009165768	2.038
DAVID	BP	GO:000728	fertilization	spermatid development	0.001993016	9.064	3.180134	0.019628187	1.707
DAVID	BP	GO:000733	fertilization	single fertilization	9.77244E-19	36.709	5.198056	1.58802E-16	15.799
DAVID	BP	GO:000734	fertilization	acrosome reaction	0.001354871	53.395	5.738624	0.014204293	1.848
DAVID	BP	GO:000956	fertilization	fertilization	8.03933E-21	33.450	5.063931	2.61278E-18	17.583
DAVID	BP	GO:001995	fertilization	sexual reproduction	3.26935E-15	8.331	3.058504	2.65635E-13	12.576
DAVID	BP	GO:002241	fertilization	cellular process involved in reproduction in multice	7.4472E-12	13.739	3.780203	2.68927E-10	9.570
DAVID	BP	GO:002241	fertilization	reproductive process	3.28857E-13	6.077	2.603325	1.33598E-11	10.874
DAVID	BP	GO:004823	fertilization	male gamete generation	0.000924832	4.887	2.288913	0.010364496	1.984
DAVID	BP	GO:004851	fertilization	spermatid differentiation	0.002274683	8.740	3.127666	0.021743289	1.663
DAVID	BP	GO:004860	fertilization	multicellular organismal reproductive process	1.48995E-13	8.270	3.047801	6.91764E-12	11.160
DAVID	BP	GO:000700	fusion of sperm to egg plasma men	plasma membrane organization	3.72735E-09	22.736	4.506894	1.00949E-07	6.996
DAVID	BP	GO:000734	fusion of sperm to egg plasma men	fusion of sperm to egg plasma membrane involved	1.5047E-15	130.520	7.028131	1.6301E-13	12.788
DAVID	BP	GO:001025	fusion of sperm to egg plasma men	endomembrane system organization	9.14083E-08	8.258	3.045771	1.98051E-06	5.703
DAVID	BP	GO:004502	fusion of sperm to egg plasma men	plasma membrane fusion	7.05811E-15	110.127	6.783018	4.58777E-13	12.338
DAVID	BP	GO:006102	fusion of sperm to egg plasma men	membrane organization	0.000452351	4.686	2.228429	0.005654387	2.248
DAVID	BP	GO:006102	fusion of sperm to egg plasma men	membrane fusion	5.82066E-09	21.488	4.425466	1.45517E-07	6.837
DAVID	BP	GO:000733	sperm-egg recognition	binding of sperm to zona pellucida	1.63594E-06	55.937	5.805738	2.95378E-05	4.530
DAVID	BP	GO:000803	sperm-egg recognition	cell recognition	8.044E-07	20.923	4.387025	1.53782E-05	4.813
DAVID	BP	GO:000998	sperm-egg recognition	cell-cell recognition	1.19885E-08	42.168	5.39808	2.78305E-07	6.555
DAVID	BP	GO:003503	sperm-egg recognition	sperm-egg recognition	1.83704E-09	57.103	5.835486	5.42763E-08	7.265
DAVID	CC	GO:000166	acrosomal vesicle	acrosomal vesicle	1.5548E-16	32.730	5.032542	6.37468E-15	14.196
DAVID	CC	GO:000208	acrosomal vesicle	acrosomal membrane	0.000110855	41.789	5.385059	0.001818024	2.740
DAVID	CC	GO:003014	acrosomal vesicle	secretory granule	5.28834E-07	5.632	2.493709	1.44548E-05	4.840
DAVID	CC	GO:009950	acrosomal vesicle	secretory vesicle	5.02992E-06	4.602	2.20231	0.000103113	3.987
DAVID	CC	GO:000557	extracellular region	extracellular region	2.03716E-27	4.206	2.072554	1.67047E-25	24.777
DAVID	CC	GO:003612	sperm flagellum	sperm flagellum	0.003141306	8.075	3.0135	0.042931176	1.367
DAVID	CC	GO:009772	sperm flagellum	9+2 motile cilium	0.004162859	7.462	2.899632	0.048764914	1.312
DAVID	MF	GO:003173	CCR chemokine receptor binding	CCR6 chemokine receptor binding	0.00930645	207.438	7.696533	0.037742824	1.423
DAVID	MF	GO:004237	CCR chemokine receptor binding	chemokine receptor binding	0.004549192	28.811	4.848536	0.020755687	1.683
DAVID	MF	GO:004802	CCR chemokine receptor binding	CCR chemokine receptor binding	0.001825328	45.758	5.515961	0.016594534	1.780
DAVID	MF	GO:000425	hydrolase activity, hydrolyzing O-gl	serine-type endopeptidase activity	0.002805867	13.647	3.770533	0.018620752	1.730
DAVID	MF	GO:000455	hydrolase activity, hydrolyzing O-gl	hydrolase activity, hydrolyzing O-glycosyl compour	5.69719E-07	35.359	5.143992	1.38632E-05	4.858
DAVID	MF	GO:000823	hydrolase activity, hydrolyzing O-gl	peptidase activity	0.003951947	5.411	2.436005	0.019655294	1.707
DAVID	MF	GO:000823	hydrolase activity, hydrolyzing O-gl	serine-type peptidase activity	0.003782468	12.274	3.617582	0.019655294	1.707
DAVID	MF	GO:001678	hydrolase activity, hydrolyzing O-gl	hydrolase activity	0.000143399	3.104	1.63414	0.002093629	2.679
DAVID	MF	GO:001679	hydrolase activity, hydrolyzing O-gl	hydrolase activity, acting on glycosyl bonds	3.51324E-06	24.500	4.614739	6.41166E-05	4.193
DAVID	MF	GO:001717	hydrolase activity, hydrolyzing O-gl	serine hydrolase activity	0.004038759	11.991	3.583833	0.019655294	1.707
DAVID	MF	GO:000379	lysozyme activity	lysozyme activity	4.05829E-12	311.156	8.281495	2.96255E-10	9.528
DAVID	MF	GO:006178	lysozyme activity	peptidoglycan muralytic activity	1.27213E-11	259.297	8.018461	4.64327E-10	9.333
DAVID	MF	GO:000486	serine-type endopeptidase inhibitor	endopeptidase inhibitor activity	0.002045901	15.253	3.930998	0.016594534	1.780
DAVID	MF	GO:000486	serine-type endopeptidase inhibitor	serine-type endopeptidase inhibitor activity	0.000562578	23.843	4.575518	0.006844702	2.165
DAVID	MF	GO:003041	serine-type endopeptidase inhibitor	peptidase inhibitor activity	0.002360049	14.506	3.85859	0.017228357	1.764
DAVID	MF	GO:004205	serine-type endopeptidase inhibitor	chemoattractant activity	0.001933705	44.451	5.47414	0.016594534	1.780
DAVID	MF	GO:006113	serine-type endopeptidase inhibitor	peptidase regulator activity	0.0058805	10.477	3.389104	0.025251557	1.598
DAVID	MF	GO:006113	serine-type endopeptidase inhibitor	endopeptidase regulator activity	0.003130085	13.129	3.71468	0.01904135	1.720
PANGEA	BF	GO:000695	defense response	defense response	5.834E-05	3.470	1.795	0.000880173	3.055
PANGEA	BF	GO:000961	defense response to bacterium	response to bacterium	4.43801E-09	8.049	3.009	1.39999E-07	6.854
PANGEA	BF	GO:004274	defense response to bacterium	defense response to bacterium	1.10871E-13	18.848	4.236	5.91882E-12	11.228
PANGEA	BF	GO:004320	defense response to bacterium	response to external biotic stimulus	1.44713E-05	3.962	1.986	0.000257514	3.589
PANGEA	BF	GO:004508	defense response to bacterium	innate immune response	0.000531109	4.232	2.081	0.005850633	2.233
PANGEA	BF	GO:005082	defense response to bacterium	defense response to Gram-negative bacterium	0.000794466	16.515	4.046	0.007990718	2.097
PANGEA	BF	GO:005083	defense response to bacterium	defense response to Gram-positive bacterium	0.001074748	14.887	3.896	0.009441455	2.025
PANGEA	BF	GO:005170	defense response to bacterium	response to other organism	1.4338E-05	3.965	1.987	0.000257514	3.589
PANGEA	BF	GO:009854	defense response to bacterium	defense response to other organism	4.53862E-07	5.420	2.438	1.21146E-05	4.917
PANGEA	BF	GO:000728	fertilization	germ cell development	0.004815197	5.848	2.548	0.033417464	1.476
PANGEA	BF	GO:000728	fertilization	spermatogenesis	0.00259231	4.297	2.103	0.020443901	1.689
PANGEA	BF	GO:000728	fertilization	spermatid development	0.000700427	9.924	3.311	0.007284776	2.138
PANGEA	BF	GO:000733	fertilization	single fertilization	2.00022E-17	36.350	5.184	3.47037E-15	14.460
PANGEA	BF	GO:000734	fertilization	acrosome reaction	1.71157E-06	45.460	5.507	4.24226E-05	4.372
PANGEA	BF	GO:000956	fertilization	fertilization	1.07562E-17	30.828	4.946	2.48827E-15	14.604
PANGEA	BF	GO:001995	fertilization	sexual reproduction	1.13059E-12	8.355	3.063	5.60451E-11	10.251
PANGEA	BF	GO:002241	fertilization	reproductive process	9.23813E-10	5.165	2.369	3.50792E-08	7.455
PANGEA	BF	GO:004823	fertilization	male gamete generation	0.003037967	4.161	2.057	0.021831985	1.661
PANGEA	BF	GO:004851	fertilization	spermatid differentiation	0.000838497	9.458	3.242	0.008082183	2.092
PANGEA	BF	GO:004860	fertilization	multicellular organismal reproductive process	0.001046987	3.814	1.931	0.009436482	2.025
PANGEA	BF	GO:000167	fusion of sperm to egg plasma men	acrosome assembly	0.001045355	41.449	5.373	0.009436482	2.025
PANGEA	BF	GO:000700	fusion of sperm to egg plasma men	plasma membrane organization	3.66483E-08	15.746	3.977	1.05975E-06	5.975
PANGEA	BF	GO:000734	fusion of sperm to egg plasma men	fusion of sperm to egg plasma membrane involved	1.09021E-13	117.438	6.876	5.91882E-12	11.228
PANGEA	BF	GO:001025	fusion of sperm to egg plasma men	endomembrane system organization	3.27696E-06	6.280	2.651	7.10691E-05	4.148
PANGEA	BF	GO:003336	fusion of sperm to egg plasma men	secretory granule organization	0.004937012	19.044	4.251	0.033923627	1.469
PANGEA	BF	GO:004502	fusion of sperm to egg plasma men	plasma membrane fusion	9.12493E-10	35.742	5.16	3.50792E-08	7.455
PANGEA	BF	GO:000237	immune response	immune system process	0.000829495	2.408	1.268	0.008082183	2.092
PANGEA	BF	GO:000695	immune response	immune response	0.000233413	3.025	1.597	0.003239779	2.489
PANGEA	BF	GO:006102	membrane fusion	membrane fusion	0.000278023	5.432	2.442	0.003640533	2.439
PANGEA	BF	GO:000733	sperm-egg recognition	binding of sperm to zona pellucida	2.21659E-06	42.705	5.416	5.12771E-05	4.290
PANGEA	BF	GO:000803	sperm-egg recognition	cell recognition	1.52035E-05	11.185	3.483	0.00026378	3.579
PANGEA	BF	GO:000998	sperm-egg recognition	cell-cell recognition	1.71911E-08	35.231	5.139	5.18721E-07	6.285
PANGEA	BF	GO:003503	sperm-egg recognition	sperm-egg recognition	1.60836E-09	51.558	5.688	5.58101E-08	7.253
PANGEA	CC	GO:000166	acrosomal vesicle	acrosomal vesicle	7.70215E-15	36.219	5.179	8.67869E-13	12.062
PANGEA	CC	GO:000208	acrosomal vesicle	acrosomal membrane	4.74122E-05	42.278	5.402	0.000731201	3.136
PANGEA	CC	GO:003014	acrosomal vesicle	secretory granule	5.59128E-06	5.244	2.391	0.000107787	3.967
PANGEA	CC	GO:009950	acrosomal vesicle	secretory vesicle	3.21993E-05	4.350	2.121	0.000519681	3.284
PANGEA	CC	GO:000557	extracellular region	extracellular region	1E-25	4.677	2.225	6.94E-23	22.159
PANGEA	CC	GO:000585	inner dynein arm	axonemal dynein complex	0.000595619	10.362	3.373	0.006359381	2.197
PANGEA	CC	GO:000587	inner dynein arm	microtubule associated complex	0.005554969	5.615	2.489	0.037114988	1.430
PANGEA	CC	GO:000587	inner dynein arm	axonemal microtubule	0.000473884	11.010	3.461	0.005481259	2.261
PANGEA	CC	GO:000588	inner dynein arm	cytoplasmic microtubule	0.002053865	7.417	2.891	0.016574212	1.781
PANGEA	CC	GO:003028	inner dynein arm	dynein complex	0.001067638	8.863	3.148	0.009441455	2.025
PANGEA	CC	GO:003615	inner dynein arm	inner dynein arm	0.000395077	11.551	3.53	0.004876028	2.312
PANGEA	CC	GO:003615	inner dynein arm	outer dynein arm	0.00048803	10.925	3.449	0.005552346	2.256
PANGEA	CC	GO:000593	sperm flagellum	axoneme	0.003000089	6.679	2.74	0.021831985	1.661
PANGEA	CC	GO:003151	sperm flagellum	motile cilium	0.002014807	7.456	2.898	0.016450303	1.784
PANGEA	CC	GO:003612	sperm flagellum	sperm flagellum	0.000248192	13.049	3.706	0.003343423	2.476
PANGEA	CC	GO:009701	sperm flagellum	ciliary plasm	0.003051445	6.647	2.733	0.021831985	1.661
PANGEA	CC	GO:009754	sperm flagellum	axonemal outer doublet	0.000407507	11.457	3.518	0.004876028	2.312
PANGEA	CC	GO:009764	sperm flagellum	A axonemal microtubule	0.000407507	11.457	3.518	0.004876028	2.312
PANGEA	CC	GO:009772	sperm flagellum	9+2 motile cilium	0.000371035	11.744	3.554	0.004768488	2.322
PANGEA	CC	GO:199071	sperm flagellum	axonemal central apparatus	0.000395077	11.551	3.53	0.004876028	2.312
PANGEA	CC	GO:009752	sperm plasma membrane	sperm plasma membrane	0.000511931	58.719	5.876	0.005730328	2.242
PANGEA	MF	GO:004237	CCR chemokine receptor binding	chemokine receptor binding	0.007233493	15.658	3.969	0.046916298	1.329
PANGEA	MF	GO:004802	CCR chemokine receptor binding	CCR chemokine receptor binding	0.002848099	25.165	4.653	0.021027451	1.677
PANGEA	MF	GO:000486	chemoattractant activity	endopeptidase inhibitor activity	0.0073382	7.550	2.916	0.047154732	1.326
PANGEA	MF	GO:004205	chemoattractant activity	chemoattractant activity	0.002649458	26.097	4.706	0.020659817	1.685
PANGEA	MF	GO:000379	lysozyme activity	lysozyme activity	3.21867E-14	234.876	7.876	2.48195E-12	11.605
PANGEA	MF	GO:000417	lysozyme activity	endopeptidase activity	0.000836111	4.517	2.175	0.008082183	2.092
PANGEA	MF	GO:000425	lysozyme activity	serine-type endopeptidase activity	3.00508E-06	11.059	3.467	6.7275E-05	4.172
PANGEA	MF	GO:000455	lysozyme activity	hydrolase activity, hydrolyzing O-glycosyl compour	1.75217E-07	24.021	4.586	4.86402E-06	5.313
PANGEA	MF	GO:000823	lysozyme activity	peptidase activity	0.000196562	4.320	2.111	0.002841953	2.546
PANGEA	MF	GO:000823	lysozyme activity	serine-type peptidase activity	4.74616E-06	10.319	3.367	9.68775E-05	4.014
PANGEA	MF	GO:001678	lysozyme activity	hydrolase activity	0.000250516	2.282	1.19	0.003343423	2.476
PANGEA	MF	GO:001679	lysozyme activity	hydrolase activity, acting on glycosyl bonds	2.19527E-06	15.658	3.969	5.12771E-05	4.290
PANGEA	MF	GO:001717	lysozyme activity	serine hydrolase activity	5.29299E-06	10.149	3.343	0.000104952	3.979
PANGEA	MF	GO:000202	protease binding	protease binding	0.001019207	6.476	2.695	0.009431062	2.025
PAN-GO	BP	GO:000733	binding of sperm to zona pellucida	binding of sperm to zona pellucida	0.00000475	87.460	6.450551	0.00228	2.642
PAN-GO	BP	GO:000998	binding of sperm to zona pellucida	cell-cell recognition	0.0000206	55.240	5.787641	0.00835	2.078
PAN-GO	BP	GO:003503	binding of sperm to zona pellucida	sperm-egg recognition	0.0000174	58.310	5.865671	0.00744	2.128
PAN-GO	BP	GO:005078	biological regulation	regulation of biological process	0.00000659	0.180	-2.473931	0.00298	2.526
PAN-GO	BP	GO:005079	biological regulation	regulation of cellular process	0.00000214	0.130	-2.943416	0.0011	2.959
PAN-GO	BP	GO:006500	biological regulation	biological regulation	0.000000915	0.160	-2.643856	0.000503	3.298
PAN-GO	BP	GO:000700	fusion of sperm to egg plasma men	plasma membrane organization	5.77E-09	41.980	5.39163	0.00000444	5.353
PAN-GO	BP	GO:000734	fusion of sperm to egg plasma men	fusion of sperm to egg plasma membrane involved	6.75E-13	> 100	6.643856	1.3E-09	8.886
PAN-GO	BP	GO:001025	fusion of sperm to egg plasma men	endomembrane system organization	0.000000127	13.390	3.743084	0.000075	4.125
PAN-GO	BP	GO:004502	fusion of sperm to egg plasma men	plasma membrane fusion	1.18E-12	> 100	6.643856	1.82E-09	8.740
PAN-GO	BP	GO:006102	fusion of sperm to egg plasma men	membrane fusion	8.79E-08	26.910	4.75007	0.0000564	4.249
PAN-GO	BP	GO:000167	single fertilization	acrosome assembly	0.000119	> 100	6.643856	0.0437	1.360
PAN-GO	BP	GO:000733	single fertilization	single fertilization	1.74E-18	> 100	6.643856	1.34E-14	13.873
PAN-GO	BP	GO:000734	single fertilization	acrosome reaction	8.77E-08	> 100	6.643856	0.0000614	4.212
PAN-GO	BP	GO:000956	single fertilization	fertilization	8.34E-18	89.710	6.487197	3.21E-14	13.493
PAN-GO	BP	GO:001995	single fertilization	sexual reproduction	8.6E-14	29.150	4.865424	2.21E-10	9.656
PAN-GO	BP	GO:002241	single fertilization	cellular process involved in reproduction in multice	8.5E-12	31.810	4.991408	1.09E-08	7.963
PAN-GO	BP	GO:002241	single fertilization	reproductive process	5.75E-11	16.100	4.008989	5.53E-08	7.257
PAN-GO	CC	GO:000166	acrosomal vesicle	acrosomal vesicle	2.68E-17	> 100	6.643856	3.68E-14	13.434
PAN-GO	CC	GO:003014	acrosomal vesicle	secretory granule	3.88E-13	44.350	5.470862	2.66E-10	9.575
PAN-GO	CC	GO:003141	acrosomal vesicle	cytoplasmic vesicle	0.00007	4.940	2.304511	0.0107	1.971
PAN-GO	CC	GO:003198	acrosomal vesicle	vesicle	0.000119	4.610	2.204767	0.0149	1.827
PAN-GO	CC	GO:009770	acrosomal vesicle	intracellular vesicle	0.0000717	4.930	2.301588	0.00985	2.007
PAN-GO	CC	GO:009950	acrosomal vesicle	secretory vesicle	7.66E-10	19.320	4.272023	0.000000351	6.455
PAN-GO	CC	GO:000557	extracellular space	extracellular region	0.00000302	4.570	2.192194	0.00083	3.081
PAN-GO	CC	GO:000561	extracellular space	extracellular space	0.00000161	4.830	2.272023	0.000552	3.258
PAN-GO	CC	GO:000562	intracellular anatomical structure	intracellular anatomical structure	0.0000131	0.400	-1.321928	0.00258	2.588
PAN-GO	CC	GO:003151	motile cilium	motile cilium	0.0000331	21.870	4.450881	0.00568	2.246
PAN-GO	CC	GO:003612	motile cilium	sperm flagellum	0.000202	26.240	4.713696	0.0231	1.636
PAN-GO	CC	GO:009772	motile cilium	9+2 motile cilium	0.000233	24.990	4.643279	0.0246	1.609
PAN-GO	CC	GO:000563	nucleus	nucleus	0.000286	0.100	-3.321928	0.028	1.553
PAN-GO	CC	GO:000219	zona pellucida receptor complex	zona pellucida receptor complex	0.00000801	> 100	6.643856	0.00183	2.738
PAN-GO	MF	GO:000455	hydrolase activity, hydrolyzing O-gl	hydrolase activity, hydrolyzing O-glycosyl compour	0.000042	20.580	4.363171	0.0223	1.652
PAN-GO	MF	GO:001679	hydrolase activity, hydrolyzing O-gl	hydrolase activity, acting on glycosyl bonds	0.0000963	16.660	4.058316	0.0438	1.359
PAN-GO	MF	GO:000379	peptidoglycan muralytic activity	lysozyme activity	4.1E-09	> 100	6.643856	0.000013	4.886
PAN-GO	MF	GO:006178	peptidoglycan muralytic activity	peptidoglycan muralytic activity	4.1E-09	> 100	6.643856	0.00000652	5.186

* BP, biological process; MF, molecular function; CC, cellular component.
† Branch of origin in the vertebrate phylogeny (Chen et al, 2025).
§ Fold enrichment, proportion of genes in the set of genes examined for a given GO term / proportion of genes in the genome background for that GO term.
¶ According to Benjamini-Hochberg (Benjamini and Holberg, 1995).

Appendix References

Benjamini Y, Hochberg Y (1995) Controlling the False Discovery Rate - a Practical and Powerful Approach to Multiple Testing. *Journal of the Royal Statistical Society Series B-Methodological* 57: 289-300

Chen JH, Landback P, Arsala D, Guzzetta A, Xia S, Atlas J, Sosa D, Zhang YE, Cheng J, Shen B *et al* (2025) Evolutionarily new genes in humans with disease phenotypes reveal functional enrichment patterns shaped by adaptive innovation and sexual selection. *Genome Res* 35: 379-392

Hu Y, Flockhart I, Vinayagam A, Bergwitz C, Berger B, Perrimon N, Mohr SE (2011) An integrative approach to ortholog prediction for disease-focused and other functional studies. *BMC Bioinformatics* 12: 357

Shao Y, Chen C, Shen H, He BZ, Yu D, Jiang S, Zhao S, Gao Z, Zhu Z, Chen X *et al* (2019) GenTree, an integrated resource for analyzing the evolution and function of primate-specific coding genes. *Genome Res* 29: 682-696
