# Supplementary material for: Male reproductive biology was reshaped during placental mammal diversification through epididymal secretome expansion
Source: EMBO Rep. 2026 Jun 3;27(13):3538–46. doi: 10.1038/s44319-026-00817-1 (PMC13354781; doi:10.1038/s44319-026-00817-1)
Supplement: Supplementary file 2 — Peer Review File [file 44319_2026_817_MOESM2_ESM.pdf]

EMBO reports

Male reproductive biology was reshaped during placental mammal diversification through epididymal secretome expansion

Jose Ranz and Alberto Civetta

Corresponding author(s): Jose Ranz (jranz@uci.edu) , Alberto Civetta (a.civetta@uwinnipeg.ca)

Review Timeline:

Submission Date:	13th Jan 26
Editorial Decision:	17th Feb 26
Revision Received:	17th Mar 26
Editorial Decision:	30th Apr 26
Revision Received:	6th May 26
Accepted:	22nd May 26

Editor: Yehu Moran

Transaction Report:

(Note: With the exception of the correction of typographical or spelling errors that could be a source of ambiguity, letters and reports are not edited. Depending on transfer agreements, referee reports obtained elsewhere may or may not be included in this compilation. Referee reports are anonymous unless the Referee chooses to sign their reports.)

Dear Prof. Ranz

Thank you for the submission of your manuscript to EMBO Reports. We have now received the full set of referee reports that are all pasted below.

As you will see, the referees acknowledge that the findings are potentially interesting. However, they do raise significant concerns regarding parts of your work that call for thorough revision and reconsideration.

I would thus like to invite you to revise your manuscript with the understanding that the referee concerns must be fully addressed and their suggestions taken on board whenever possible. Please address all referee concerns in a complete point-by-point response. Acceptance of the manuscript will depend on a positive outcome of a second round of review. It is EMBO Reports policy to allow a single round of major revision only and acceptance or rejection of the manuscript will therefore depend on the completeness of your responses included in the next version of the manuscript.

We realize that it is difficult to revise to a specific deadline. In the interest of protecting the conceptual advance provided by the work, we recommend a revision within 3 months (20th May 2026). Please discuss the revision progress ahead of this time with the editor if you require more time to complete the revisions.

You can either publish the study as a short report or as a full article. For short reports, the revised manuscript should not exceed 27,000 characters (including spaces but excluding materials & methods and references) and 5 main plus 5 expanded view figures. The results and discussion sections must further be combined, which will help to shorten the manuscript text by eliminating some redundancy that is inevitable when discussing the same experiments twice. For a normal article there are no length limitations, but it should have more than 5 main figures and the results and discussion sections must be separate. In both cases, the entire materials and methods must be included in the main manuscript file.

Yet, please note that in light of the nature of your work I would strongly lean towards a short report format that I believe suits it much better.

IMPORTANT NOTE: we perform an initial quality control of all revised manuscripts before re-review. Your manuscript will FAIL this control and the handling will be DELAYED if the following APPLIES:
1) A data availability section providing access to data deposited in public databases is missing. If you have not deposited any data, please add a sentence to the data availability section that explains that.
2) Your manuscript contains statistics and error bars based on n=2. Please use scatter blots in these cases. No statistics should be calculated if n=2.

When submitting your revised manuscript, please review the submission guidelines (https://link.springer.com/journal/44319/submission-guidelines#cms-Revised-submissions) and carefully review the instructions that follow below. Failure to include requested items will delay the evaluation of your revision.

1) a .docx formatted version of the manuscript text (including legends for main figures, EV figures and tables). Please make sure that the changes are highlighted to be clearly visible.

2) individual production quality figure files as .eps, .tif, .jpg (one file per figure). See hhttps://media.springernature.com/original/springer-cms/rest/v1/content/27825798/data/v1 for more info on how to prepare your figures.

3) We replaced Supplementary Information with Expanded View (EV) Figures and Tables that are collapsible/expandable online. A maximum of 5 EV Figures can be typeset. EV Figures should be cited as 'Figure EV1, Figure EV2" etc... in the text and their respective legends should be included in the main text after the legends of regular figures.

- For the figures that you do NOT wish to display as Expanded View figures, they should be bundled together with their legends in a single PDF file called *Appendix*, which should start with a short Table of Content. Appendix figures should be referred to in the main text as: "Appendix Figure S1, Appendix Figure S2" etc. See 'Expanded View' section of the submission guidelines.

- Additional Tables/Datasets should be labeled and referred to as Table EV1, Dataset EV1, etc. Legends have to be provided in a separate tab in case of .xls files. Alternatively, the legend can be supplied as a separate text file (README) and zipped together with the Table/Dataset file.

4) a .docx formatted letter INCLUDING the reviewers' reports and your detailed point-by-point responses to their comments. As part of the EMBO Press transparent editorial process, the point-by-point response is part of the Review Process File (RPF), which will be published alongside your paper.

5) a complete author checklist, which you can download from our author guidelines. Please insert information in the checklist that is also reflected in the manuscript. The completed author checklist will also be part of the RPF.

6) Please note that all corresponding authors are required to supply an ORCID ID for their name upon submission of a revised manuscript (<https://orcid.org/>), which can be linked to your profile in the manuscript submission system.

7) Before submitting your revision, primary datasets produced in this study need to be deposited in an appropriate public database. Please remember to provide a reviewer password if the datasets are not yet public. The accession numbers and database should be listed in a formal "Data Availability" section placed after Methods. Please note that the Data Availability Section is restricted to new primary data that are part of this study. * Note - All links should resolve to a page where the data can be accessed. *

If your study has not produced novel datasets, please mention this fact in the Data Availability Section.

8) At EMBO Press we ask authors to provide source data for the main manuscript figures. You will receive a separate email with instructions for providing source data with your revised manuscript, including how to upload and organize the files.

9) Our journal also encourages inclusion of *data citations in the reference list* to directly cite datasets that were re-used and obtained from public databases. Data citations in the article text are distinct from normal bibliographical citations and should directly link to the database records from which the data can be accessed. In the main text, data citations are formatted as follows: "Data ref: Smith et al, 2001" or "Data ref: NCBI Sequence Read Archive PRJNA342805, 2017". In the Reference list, data citations must be labeled with "[DATASET]". A data reference must provide the database name, accession number/identifiers and a resolvable link to the landing page from which the data can be accessed at the end of the reference.

10) Regarding data quantification, the following points must be specified in each figure legend:

- the name of the statistical test used to generate error bars and P values,

- the number (n) of independent experiments (please specify technical or biological replicates) underlying each data point,

- the nature of the bars and error bars (s.d., s.e.m.),

- If the data are obtained from n Program fragment delivered error ``Can't locate object method "less" via package "than" (perhaps you forgot to load "than"?) at //ejpvfs23/sites23b/embor_www/letters/embor_decision_revise_and_review.txt line 54.' 2, use scatter blots showing the individual data points.

Discussion of statistical methodology can be reported in the materials and methods section, but figure legends should contain a basic description of n, P and the test applied.

- Please also include scale bars in all microscopy images.

11) The journal requires a statement specifying whether or not authors have competing interests (defined as all potential or actual interests that could be perceived to influence the presentation or interpretation of an article). In case of competing interests, this must be specified in your disclosure statement.

12) All Materials and Methods need to be described in the main text using our 'Structured Methods' format, which is required for all research articles. According to this format, the Methods section includes a Reagents and Tools Table (listing key reagents, experimental models, software and relevant equipment and including their sources and relevant identifiers) followed by a Methods and Protocols section describing the methods using a step-by-step protocol format. The aim is to facilitate adoption of the methodologies across labs. More information on how to adhere to this format as well as a downloadable template (.docx) for the Reagents and Tools Table can be found in our author guidelines.

We would also welcome the submission of cover suggestions, or motifs to be used by our Graphics Illustrator in designing a cover.

As part of the EMBO publication's Transparent Editorial Process, EMBO reports publishes online a Review Process File (RPF) to accompany accepted manuscripts. This File will be published in conjunction with your paper and will include the referee reports, your point-by-point response and all pertinent correspondence relating to the manuscript.

You are able to opt out of this by letting the editorial office know (emboreports@embo.org). If you do opt out, the Review Process File link will point to the following statement: "No Review Process File is available with this article, as the authors have chosen not to make the review process public in this case."

I look forward to seeing a revised form of your manuscript when it is ready.

Yours sincerely,

Yehu Moran
Academic Editor
EMBO Reports

Referee #1:

Jose M. Ranz and Alberto Civetta submitted the manuscript titled "Male reproductive biology was reshaped during placental mammal diversification through epididymal secretome expansion." The central idea is interesting and potentially important, with a meaningful signal in the data. However, the current version quickly moves from annotation patterns in human datasets to broad claims about evolutionary events and biological reconfiguration across vertebrates. The analyses do not yet justify claims of large-scale evolutionary reshaping of male reproductive biology. The manuscript needs substantial revision to support these conclusions convincingly. If the journal prioritizes rigorous evolutionary inference over annotation-driven patterns centered on humans, the study may need significant revisions.
Major comments:
1) The inference of gene "origin" at specific phylogenetic branches can be distorted by orthology errors, gene loss, rapid divergence, or annotation gaps, inflating apparent lineage-specific gains.
2) The analysis uses human tissue-specific expression data to support vertebrate evolutionary conclusions, without cross-species validation of expression or function conservation.
3) The definition and construction of the "male reproductive secretome" lack sufficient methodological clarity and sensitivity testing to rule out artifacts.
4) The statistical framework, including enrichment calculations and simulations, needs clearer justification and reporting to show robustness and proper control for multiple testing.
5) Functional interpretations rely on select genes, not comprehensive evidence that the whole gene set caused a biological shift.
6) The manuscript overstates its conclusions, citing annotation-based enrichment as evidence of evolutionary reconfiguration without enough comparative or mechanistic support.

Referee #2:

Summary:
In this manuscript, Ranz & Civetta present an ambitious and conceptually compelling computational evolutionary genomics study that leverages curated human secretome annotations from the Human Protein Atlas together with phylogenetic gene-age assignments to investigate the evolutionary timing of secretory proteins involved in male reproduction. The authors provide evidence for a pronounced expansion of the male reproductive secretome during the eutherian radiation, with a particularly strong enrichment for epididymis-expressed genes involved in post-mating processes.

The study is clearly written, analytically rigorous, and presents a coherent single message that is well aligned with the scope and format of EMBO reports. The statistical framework is appropriate, and the conclusions are generally supported by the data. Overall, I find the manuscript strong and of broad interest. My comments below are largely aimed at improving contextualization and biological framing rather than requesting additional analyses.

EMBO Questions
1. Does this manuscript report a single key finding?
Yes - The male reproductive secretome underwent a uniquely large expansion during the eutherian radiation, driven primarily by epididymis-expressed genes associated with post-mating reproductive processes.

2. Is the reported work of significance?
Yes.

3. Is it of general interest to the molecular biology community?
Yes - The work connects evolutionary genomics with reproductive biology and secretome function, providing a conceptual framework for understanding how post-mating reproductive biology was reshaped during placental mammal diversification.

4. Is the major finding robustly documented using independent lines of evidence?
YES - within the scope of a computational evolutionary study.

Major Comments:

1. Integration of epididymal biology literature. While the manuscript makes a strong case for the central role of the epididymis in the evolutionary reconfiguration of the male reproductive secretome, important literature from the epididymal biology field is not currently cited. I appreciate that not all relevant work can be included, however, incorporation of a small number of key studies would substantially strengthen the biological context and help anchor the evolutionary findings within contemporary epididymal physiology. Some examples of relevant literature include:
• Gervasi & Visconti, 2017, Andrology
• Nixon et al., 2020, Molecular and Cellular Endocrinology
• Skerrett-Byrne et al., 2022, Cell Reports
• Teves & Roldan 2022 Physiol Rev
These studies would reinforce the argument that the epididymis is not merely a conduit but a highly dynamic regulatory organ whose molecular complexity aligns well with the authors' evolutionary conclusions.

2. Explicit consideration of extracellular vesicles and epididymosomes. Given that this manuscript focuses on the secretome of the male reproductive tract, it is notable that extracellular vesicles, particularly epididymosomes, are not discussed. Epididymosomes are now well established as a major mode of protein and RNA delivery to sperm and represent a key functional component of the epididymal secretome. While I am not strictly suggesting that the authors perform new analyses, the Discussion would benefit from acknowledging this secretory route and its relevance to the interpretation of the findings. Relevant studies include:
• Yuan et al., 2013, PLoS One
• Battistone et al., 2019, Journal of Cell Science
• Nixon et al., 2019, Molecular & Cellular Proteomics
• Chan et al., 2020, Nature Communications
• Tamessar et al., 2025, Proteomics
Incorporating epididymosomes into the conceptual framework would strengthen the link between secretome expansion and functional delivery mechanisms.

3. Incorporation of recent integrative epididymal proteomics resources. The authors may not be aware of a very recent publication in Reproduction (PMID: 41575304) that integrates all publicly available epididymal proteomics datasets into an accessible resource (https://reproproteomics.shinyapps.io/ShinyEpididymis/). This platform allows rapid querying of protein presence across tissues, sperm, and epididymosomes.
A cursory examination of genes highlighted in the present manuscript (e.g., SPINT4, GPX5, GPX6) confirms their presence and even gradient expression across sperm, epididymosomes, and/or epididymal tissue, providing independent proteomic support for the authors' conclusions. Referencing this resource would strengthen the manuscript without requiring additional analyses.

4. Broader framing of epididymal secretions and paternal epigenetic inheritance. Beyond sperm function per se, the findings prompt consideration of emerging literature showing that epididymal secretions, particularly epididymsome cargo, contribute to shaping of the sperm epigenome and influencing offspring phenotypes. This post-testicular window has been proposed as a rapid and potentially adaptive interface between paternal environment and the next generation. While speculative framing should remain measured, a brief acknowledgment of this literature could enhance the conceptual impact of the study. Relevant references include:
• Sharma et al., 2018, Developmental Cell
• Conine et al., 2018, Developmental Cell
• Trigg et al., 2019, Cell Reports
• Spadafora, 2023, Progress in Biophysics and Molecular Biology
• Trigg & Schjenken et al., 2024, PNAS
• Skerrett-Byrne et al., 2025, Molecular Nutrition & Food Research
This addition would help situate the evolutionary expansion of the epididymal secretome within a modern framework of paternal effects and intergenerational biology.

Referee #3:

This manuscript investigates the evolutionary dynamics of the human secretome by integrating Human Protein Atlas (HPA) annotations with vertebrate gene-age datasets (Chen et al. 2025 and GenTree). The authors report a punctuated expansion of the male reproductive (MR) secretome at the eutherian branch (Branch 5, Figs. 1, 2), specifically linked to epididymal enrichment and post-mating functions (Fig. 3). The study proposes that the epididymis acted as a "maturation laboratory" for sperm during the radiation of placental mammals, shifting from structural roles to regulatory and immune-related functions.
The conclusion that the epididymal/MR secretome underwent a major expansion during placental mammal diversification is novel and compelling, and aligns with current models of reproductive systems as hotspots for adaptive innovation. The integration of gene dating data with expression profile is methodologically sound, and routinely performed in the field of new gene origination. Thus, in principle, I support the publication of this manuscript. However, I have a few major or minor comments, which serves to further improve the manuscript.

Major concerns
1. Since marsupials also possess an epididymis and undergo sperm maturation, I suggest that the authors discuss or analyze whether the eutherian secretome contains specific functional classes that are truly absent in theria (Branch 4), and whether these may be linked to placental-specific selective pressures. For example, why did particular glutathione peroxidases such as GPX5 and GPX6 evolve only in eutherians? Alternatively, could the expansion of the MR secretome have occurred independently in the marsupial lineage?
2. In line 101, the authors state that the expansion is independent of multigene family proliferation. I am curious how the MR secretome at Branch 5 arises under this scenario. Do these genes represent potential de novo origins (i.e., genes without paralogs predating Branch 5)? If so, how many such genes are there?

Minor concerns
1. The study relies on human gene age and expression data to define "epididymal enrichment" in eutherian mammals. The manuscript would be substantially strengthened if the authors could reproduce this pattern using gene age and expression data from another mammalian lineage (e.g., mouse). While GenTree provides mouse gene age data, I am unsure whether comparable HPA-like expression datasets are available for mouse. If such data are not readily accessible, or if the required analyses would be overly time-consuming, the authors could at least discuss the possibility of testing this pattern in other mammalian lineages in future work.
2. Some typos are present in figures.
o Figure 2b Legend: Correction needed: "paralago" should be "paralog".
o Figure 3a: Please clarify why 176 testis-enriched genes are not classified within the MR secretome, and delete "Encoding" in the Y-axis label.
o Figure 3c: In Y-axis legend, "Number"=>"Number of"
3. In line 74, the number 2510 appears inconsistent with the previously mentioned 2520. Please verify if this is a typo or the result of a specific filter.

Referee #1:

Jose M. Ranz and Alberto Civetta submitted the manuscript titled "Male reproductive biology was reshaped during placental mammal diversification through epididymal secretome expansion." The central idea is interesting and potentially important, with a meaningful signal in the data. However, the current version quickly moves from annotation patterns in human datasets to broad claims about evolutionary events and biological reconfiguration across vertebrates. The analyses do not yet justify claims of large-scale evolutionary reshaping of male reproductive biology. The manuscript needs substantial revision to support these conclusions convincingly. If the journal prioritizes rigorous evolutionary inference over annotation-driven patterns centered on humans, the study may need significant revisions.

We appreciate the comments of the reviewer, but we respectfully disagree with them in many respects as we will elaborate further.

Major comments:

1) The inference of gene "origin" at specific phylogenetic branches can be distorted by orthology errors, gene loss, rapid divergence, or annotation gaps, inflating apparent lineage-specific gains.

Any comparative study has associated an error rate in terms of orthologous calls, which should be higher for ancient than for recently originated genes. This is because the ability to detect sequence similarity and synteny decreases with divergence over evolutionary time. This effect would be expected to be more accentuated among rapidly diverging genes, like those associated with reproduction or immunity. Thus, any bias would most likely fail to detect ancient orthologs among rapidly evolving genes, which is not what we find in our study. Our work is neither based on a handful of genes, nor concludes that the evolutionary transition associated with primates is the one gaining proportionally more genes. Our work considers 2520 genes, ~13% of the annotated protein-coding gene complement of the human genome, and on a rigorous orthology examination by eminent colleagues that has already passed the peer-reviewed filter. This information can be found in two publications

- Shao et al. 2019 Genome Research (https://genome.cshlp.org/content/29/4/682)

For details about the impact of this impressive paper, please see its assessment in Dimensions: https://badge.dimensions.ai/details/id/pub.1112708781.

- Chen et al. 2025 Genome Research (https://genome.cshlp.org/content/35/3/379)

For details about the impact of this recent paper, please see its assessment in Dimensions: https://badge.dimensions.ai/details/id/pub.1185567065.

The paper by Shao et al. 2019, and its update (Chen et al. 2025), deal quite well with the doubts indicated by the reviewer. For example, the genomes used by Shao et al. reached the assembly level of chromosome for 18 species, with 3 cases reaching the assembly level of scaffold, and in only two cases with the assembly level of contig. Even if one would suspect bias introduced by completeness of genome assemblies, thus favoring certain branches over others, such bias should also be expected to equally impact multiple secretomes and not just the MRS.

Equally important, we do not see what leads the reviewer to think that the significant, dramatic gene-gain we document for the MR secretome by branch 5 combination is preferentially impacted by the potential problems listed in their remark. Why should not annotation errors impact other secretome by branches combinations as well? When considering the number of genes we analyzed, any error rate should be randomly distributed across the 70 secretome by phylogenetic branch combinations considered. And the importance of the effect detected is not limited to a significant *p*-adjusted value but also illustrated by the size effect we report (6.8-fold gene gain). All these aspects, when considered together, are difficult to reconcile with this type of generic and maximalist criticisms.

2) The analysis uses human tissue-specific expression data to support vertebrate evolutionary conclusions, without cross-species validation of expression or function conservation.

The request by the reviewer falls more in the realm of what would be ideal than what can be done/analyzed realistically at this moment. The functional data we use comes from, to our knowledge, the most comprehensive tissue-based analysis ever done at the transcriptomic and proteomic levels in any animal species, i.e. humans:

● Uhlén et al. 2019 Science Signaling (https://www.science.org/doi/10.1126/scisignal.aaz0274)

● Uhlén et al. 2015 Science (https://www.science.org/doi/10.1126/science.1260419)

For details about the impact of these impressive papers, please see their assessment in Dimensions:

https://badge.dimensions.ai/details/id/pub.1122914503

and

https://badge.dimensions.ai/details/id/pub.1048221082.

To best of our knowledge, there is no other vertebrate species with this wealth of functional data, not alone when we think about the different secretomes in the organism. The data does not exist.

Further, the approach adopted is commonplace in the literature. For example, *D. melanogaster* is the focal species in the genus *Drosophila* and across Diptera, with functional data being abundant in the focal species and to a much lesser extent in a few other *Drosophila* and mosquito species, primarily at the transcriptomic level. Unless specific assays are done in a different Diptera species, the information in *D. melanogaster* is extrapolated to other Diptera species, admittedly assuming some error rate. Likewise, the information from model organisms, specifically the functional annotation of their genes, is used to gain insights about homologous genes in non-model species. That is at the core of the myriads of analyses that are published every week across journals in which the authors use tools that rely in Gene Ontology functional rubrics and KEGG pathways such as DAVID, Panther, g:Profiler to name a few.

We have, based on a concrete suggestion from referee #2, added proteomics data from mice from a very recent (February 2026) publication in the journal Reproduction (PMID: 41575304) that integrates epididymal proteomics datasets into an accessible resource (https://reproproteomics.shinyapps.io/ShinyEpididymis/). Using this resource, we cross-validate an important key human finding of epididymis expression enrichment along the eutherian branch, with most of these genes (19 out of 25) also being expressed in the mouse epididymis.

Also, note that some of the genes we discuss in order to illustrate main functional enrichment patterns have been silenced by other colleagues in rodents, providing legitimacy to the extrapolation from humans to other eutherians. This is the case for *CES5A, SPINT4, Wfdc* genes, and *GPX5* to name some relevant cases.

3) The definition and construction of the "male reproductive secretome" lack sufficient methodological clarity and sensitivity testing to rule out artifacts.

We must dissent from the reviewer’s opinion. We use the secretome categorization already established in the Human Proteome Atlas database, where it is possible to find very precise information about how the different secretomes were delineated. This database is based largely on the two publications highlighted in point 2, which are very transparent and detailed in how the data were generated and handled. Additionally, the reviewer can find information about the criteria used to identify secreted proteins here:

https://www.proteinatlas.org/humanproteome/tissue/secretome

And, in figure 1, within that link, the reviewer can explore the different secretomes (composition and characteristics), including information about the origin of the proteins locally secreted in different tissues.

In other words, the categorization in terms of tissue of origin was done rigorously based on expression data within a quantitative framework. We therefore do not have anything to object to the inferences made by the authors of the two highlighted papers, and we feel reassured in using their data.

4) The statistical framework, including enrichment calculations and simulations, needs clearer justification and reporting to show robustness and proper control for multiple testing.

We prove our point about a dramatic gene gain in the MRS at branch 5 using two complementary statistical approaches. The first is a conventional test of independence via a chi-squared contingency table analysis followed by the analysis of the residuals. Each residual has an associated p-value, which is corrected for multiple testing using Benjamini-Hochberg. In the Methods section, lines 290-293 of the resubmission, we say:

“*Test for equality of proportions, two-tailed Fisher’s exact test, χ^2 test of independence, analysis of residuals, and the Benjamini–Hochberg correction for multiple testing were conducted using built-in functions in R (R Development Core Team, 2016). In the case of χ^2 tests, the null hypothesis was tested by performing 2000 Monte Carlo simulations.”*

And in the legend of Fig. 1, we say:

"Green shaded counts represent cases of statistical enrichment at 1% FDR, according to a post-hoc residual analysis following a χ^2 test of independence (Table S1)."

The tests described above are used by many and should suffice to back up our results, particularly given the use of a stringent 1% threshold after FRD corrections for multiple tests. Nevertheless, we take a second statistical approach (Monte Carlo simulations) trying to overcome two issues associated with the chi-squared test:

1. The use of a metric (Enrichment Score or ES) that conveys more intuitively the finding of gene gain compared to the absolute value of the residual.

2. Avoid the disproportionate effect that smaller branches might have as it is discussed in the pertinent section of the Methods.

To be precise, this is the section (lines 249-273) we are referring to:

"The enrichment score ($ES_{i,j}$) quantifies the fold gain of new genes for a particular secretome (i) by branch (j) combination relative to the random expectation as:

$$ES_{i,j} = \frac{Proportion\ of\ gained\ genes\ by\ secretome\ i\ in\ branch\ j}{Overall\ proportion\ of\ gained\ genes\ in\ branch\ j} = \frac{\frac{n_{i,j}}{N_i}}{\frac{N_j}{T}}$$

where i and j can be any combination of the 10 secretomes and seven phylogenetic branches, respectively (see Main Text). The variable $n_{i,j}$ corresponds to the number of gained genes by secretome i in branch j, N_i to the total number of gained genes by the secretome i, N_j to the total number of gained genes by branch j, and T to the total number of genes considered across branches, i.e., 19,665. An ES value of 1 indicates no departure from random expectation, while values >1 or <1 indicate that more or fewer genes than expected, respectively, were gained for a given secretome by branch combination. Statistical significance of ES values was assessed through Monte Carlo simulations (n=100,000) using an in-house R script (Ranz & Civetta, 2026). Simulations resampled genes without replacement under the assumption that genes are randomly distributed across secretomes and branches, with the constraint that each branch maintains a fixed number of genes (Chen et al, 2025; Shao et al, 2019). From these simulations, we calculated the 99% confidence interval (CI) and the mean simulated ES for each secretome by branch combination. Raw p-values for the 70 ES values, one for each secretome by branch combination, were corrected for multiple testing (Benjamini & Hochberg, 1995). The ES metric implements normalization by branch size, defined as the fraction of genes originating at a given branch relative to the whole genome. Consequently, ES is more sensitive to deviations occurring in smaller branches compared to larger ones. In comparison, the χ^2 test of independence identifies the largest absolute departures between observed and expected gene counts for each secretome-branch combination relative to the proportion of genes in the entire genome originating at that same branch and secretome. However, unlike the χ^2 test, ES accounts for differential branch sizes through its normalization approach."

We consider that we are very explicit, transparent, and rigorous about how we perform the analysis while indicating that the p-value associated with each of the ES (there are 70, one per secretome by branch combination) is corrected using Benjamini-Hochberg. Notice that we are performing 100,000 simulations, which are used to calculate not only the p-value for each ES but also their 99% confidence intervals (Fig. 2). Additionally, we share our code through Zenodo (https://zenodo.org/records/18157698), so the reviewer can check our R code. This is the key section of our script (in yellow):

```
# ============================================================
# MULTIPLE TESTING CORRECTION (Benjamini-Hochberg FDR)
# ============================================================
cat("\nApplying Benjamini-Hochberg FDR correction for multiple testing...\n")
# Apply Benjamini-Hochberg correction
p_values_vector <- as.vector(p_value_matrix)
adjusted_p_values <- p.adjust(p_values_vector, method = "BH")
adjusted_p_matrix <- matrix(adjusted_p_values, nrow = nrow(p_value_matrix), ncol = ncol(p_value_matrix))
rownames(adjusted_p_matrix) <- rownames(p_value_matrix)
colnames(adjusted_p_matrix) <- colnames(p_value_matrix)
```

To sum up, we used a conventional statistical test, and a second proposed approach that includes Monte Carlo simulations. In both cases, whatever p-value we obtain is subject to correction for multiple testing (Benjamini-Hochberg), always adopting a 1%FDR. The outcome of both approaches support that the gene gain experienced by the MRS at branch 5 is unique. The perfect agreement between both approaches underscores even more the strength and reliability of our findings.

5) Functional interpretations rely on select genes, not comprehensive evidence that the whole gene set caused a biological shift.

Again, we must disagree with the reviewer. We are very clear in the text (lines 138-141) that we performed functional enrichment analyses considering multiple genes (see bolded):

"We used Gene Ontology (GO) terms to identify gene functions and determine enriched categories ***among the gained genes during the eutherian radiation****. To minimize biases from relying on a single functional annotation source, we integrated results from three tools (DAVID, PANGEA, and PAN-GO) (Methods; Appendix Table S5)."*

Notice that to prevent any bias by the information present in a given database, we used three different tools that differ to some extent in their underlying algorithms, and then we integrate the outcomes. We believe that this is unusual while speaking about our efforts to generate reliable outcomes.

Lastly, once the main global patterns are presented (Fig. 3b-c), we move to an integration of the GO enrichment patterns and expression data, focusing on several examples that illustrate important aspects. We discuss some remarkable cases that have been studied in great detail, including functional assays, mostly in mouse to further prove the relevance of these genes across eutheria.

6) The manuscript overstates its conclusions, citing annotation-based enrichment as evidence of evolutionary reconfiguration without enough comparative or mechanistic support.

We would wish that equivalent proteomic and functional data exists in several other species among those considered in our article but unfortunately it is not the case. Nevertheless, our work should prompt and encourage the generation of equivalent datasets in other organisms, contributing to some sort of Earth BioGenome Project but at the functional-tissue level. We therefore align with the reviewer in cautioning the readers about the need to have equivalent data to that in the Human Proteome Atlas in other species, which is now incorporated into the last paragraph. Specifically, we say (lines 221-225):

"*Nevertheless, a detailed understanding about the evolutionary sequence of molecular changes underlying such overhaul, as well as commonalities and more lineage-restricted changes in epididymis function, will only be possible once systematic transcriptomic and proteomic analyses of the epididymis are performed across the vertebrate phylogeny*."

Relative to the last bit ("…without enough comparative or mechanistic support."), we can only say that the comparative analyses that generated the datasets we use, plus the functional data (proteomic and transcriptomic) considered, are part of outstanding peer-reviewed publications. We acknowledge that we do not perform directly any gene silencing experiment, but we cite -and minimally discuss- some relevant ones that are part of single-gene (or a few related genes)-focused papers, where reproductive phenotypes were thoroughly examined:

- *SPINT4,* Robertson *et al*, 2020, *BMC Biol*
- *Wfdc9, Wfdc11*, and *Wfdc13,* Kent et al, 2024*, PNAS*
- *GPX5,* Chabory et al, 2009*, J Clin Invest*
- *CES5A*, Ru et al, 2015, *Asian J Androl*

Lastly, the journal webpage (https://link.springer.com/journal/44319) states: *Embo Reports publishes "Short and sweet' research reports - a single key finding, backed up by multiple lines of experimental evidence.* ***Comprehensive mechanistic insight is not required****."*.

Referee #2:

Summary:

In this manuscript, Ranz & Civetta present an ambitious and conceptually compelling computational evolutionary genomics study that leverages curated human secretome annotations from the Human Protein Atlas together with phylogenetic gene-age assignments to investigate the evolutionary timing of secretory proteins involved in male reproduction. The authors provide evidence for a pronounced expansion of the male reproductive secretome during the eutherian radiation, with a particularly strong enrichment for epididymis-expressed genes involved in post-mating processes.

The study is clearly written, analytically rigorous, and presents a coherent single message that is well aligned with the scope and format of EMBO reports. The statistical framework is appropriate, and the conclusions are generally supported by the data. Overall, I find the manuscript strong and of broad interest. My comments below are largely aimed at improving contextualization and biological framing rather than requesting additional analyses.

EMBO Questions

1. Does this manuscript report a single key finding?

Yes - The male reproductive secretome underwent a uniquely large expansion during the eutherian radiation, driven primarily by epididymis-expressed genes associated with post-mating reproductive processes.

2. Is the reported work of significance?

Yes.

3. Is it of general interest to the molecular biology community?

Yes - The work connects evolutionary genomics with reproductive biology and secretome function, providing a conceptual framework for understanding how post-mating reproductive biology was reshaped during placental mammal diversification.

4. Is the major finding robustly documented using independent lines of evidence?

YES - within the scope of a computational evolutionary study.

We thank the encouraging evaluation of our manuscript by the reviewer.

Major Comments:

1. Integration of epididymal biology literature. While the manuscript makes a strong case for the central role of the epididymis in the evolutionary reconfiguration of the male reproductive secretome, important literature from the epididymal biology field is not currently cited. I appreciate that not all relevant work can be included, however, incorporation of a small number of key studies would substantially strengthen the biological context and help anchor the evolutionary findings within contemporary epididymal physiology. Some examples of relevant literature include:

• Gervasi & Visconti, 2017, Andrology

• Nixon et al., 2020, Molecular and Cellular Endocrinology

• Skerrett-Byrne et al., 2022, Cell Reports

• Teves & Roldan 2022 Physiol Rev

These studies would reinforce the argument that the epididymis is not merely a conduit but a highly dynamic regulatory organ whose molecular complexity aligns well with the authors' evolutionary conclusions.

We fully agree with the reviewer that we can do much better in highlighting the complexity of role exerted by the epididymis. According to this, we have added a sentence that captures this unappreciated complexity by many. It reads (lines 134-137):

“Moreover, the epididymis contributes to sperm structural diversity and to a major molecular remodeling that involves lipid, epigenetic and proteome changes associated with sperm maturation (Nixon et al, 2019a; Skerrett-Byrne et al, 2022; Teves & Roldan, 2022).”

2. Explicit consideration of extracellular vesicles and epididymosomes. Given that this manuscript focuses on the secretome of the male reproductive tract, it is notable that extracellular vesicles, particularly epididymosomes, are not discussed. Epididymosomes are now well established as a major mode of protein and RNA delivery to sperm and represent a key functional component of the epididymal secretome. While I am not strictly suggesting that the authors perform new analyses, the Discussion would benefit from acknowledging this secretory route and its relevance to the interpretation of the findings. Relevant studies include:

• Yuan et al., 2013, PLoS One

• Battistone et al., 2019, Journal of Cell Science

• Nixon et al., 2019, Molecular & Cellular Proteomics

• Chan et al., 2020, Nature Communications

• Tamessar et al., 2025, Proteomics

Incorporating epididymosomes into the conceptual framework would strengthen the link between secretome expansion and functional delivery mechanisms.

We agree with the reviewer as in fact we find that 7 out 9 human genes enriched in expression in epididymis with orthologs in mouse originated at branch 5, i.e. during the eutherian radiation, and expressed in the mouse epididymosomes.

We have now incorporated a short paragraph focused on the indicated results while highlighting the importance of epididymosomes. The new paragraph now reads (lines 190-199):

"Another interesting trend emerges when analyzing the presence of the MR secretome among the orthologous proteins in the mouse epididymis. An important secretory mode of protein and RNA delivery to sperm takes place within the epididymis via epididymosomes, which are key for sperm maturation and transgenerational paternal transmission of information (Chan et al, 2020; Nixon et al, 2019b). Among the nine genes with an epididymis enriched expression profile in humans that have evidence of having an ortholog among 12 proteins found in mouse epididymosomes (Trigg et al., 2026), there are seven that originated at branch 5 (CES5A, CLPSL2, GPX5, GPX6, LCN8, RNASE9, and SPINT4; CRISP1 and RNASE12 originated in earlier branches). This finding suggests that the delivery of molecules by extracellular vesicles that influence pregnancy and offspring fitness was also impacted during the eutherian radiation."

3. Incorporation of recent integrative epididymal proteomics resources. The authors may not be aware of a very recent publication in Reproduction (PMID: 41575304) that integrates all publicly available epididymal proteomics datasets into an accessible resource (https://reproproteomics.shinyapps.io/ShinyEpididymis/). This platform allows rapid querying of protein presence across tissues, sperm, and epididymosomes.

A cursory examination of genes highlighted in the present manuscript (e.g., SPINT4, GPX5, GPX6) confirms their presence and even gradient expression across sperm, epididymosomes, and/or epididymal tissue, providing independent proteomic support for the authors' conclusions. Referencing this resource would strengthen the manuscript without requiring additional analyses.

We sincerely appreciate the information provided by the reviewer. We checked for the presence of genes with enriched expression in epididymis, according to the Human Proteome Atlas, in ShinyEpididymis. For the 61 genes with enriched expression in epididymis (Fig. 3a), we have found 25 (41%), which is very encouraging. What is more reassuring is what we found when we considered the information for all 104 genes with tissue-enriched expression within the MR secretome. This was done considering different patterns of human tissue expression enrichment:

	tes_epi_pro_sem	Branch 0-4, 6	Branch 5	
Expression Profile (HUMAN PROT ATLAS)	1_0_0_0	18	10	28
	0_1_0_0	6	19	25
	0_0_1_0	0	0	0
	0_0_0_1	0	0	0
		24	29	53

One can see that among the genes found in ShinyEpididymis, those with enrichment in testis according to the Human Protein Atlas (1_0_0_0), the prevalent origin is at any branch but at 5 (18 out of 28), whereas among those with enrichment in epididymis (0_1_0_0) the prevalent origin is at branch 5 (19 out 25). Performing a two-tailed Fisher's exact test shows that there is a non-random association between branch of origin and expression profile:

```
----------------------------------------
 TABLE = [ 18 , 10 , 6 , 19 ]
Left   : p-value = 0.9994749860406079
Right  : p-value = 0.0035074803542195655
2-Tail : p-value = 0.005407066410547486
----------------------------------------
```

We believe that the finding, despite important methodological differences between how the data in the Human protein Atlas and ShinyEpididymis were generated, increases the robustness of our conclusions. This new analysis has been incorporated briefly in the existing text, including a new supplementary table (Table S4). The text (lines 119-128), with the added bit relating to the analysis using data from ShinyEpididymis (see yellow below), now reads:

The direct contrast between enriched expression in testis versus epididymis confirmed that testis-enriched expression was disproportionately linked to genes originated in branches other than branch 5 (24/36), while epididymis-enriched expression was linked to genes originated in branch 5 (43/61; two-tailed Fishers exact test, 2TFET, P = 6.0×10^{-4}) (Fig. 3a). This finding was reproduced when only genes with an ortholog expressed in the mouse epididymis (Trigg et al, 2026) were considered (19/25 vs 10/28; 2TFET, P = 5.4×10^{-3}) (Table S4). Furthermore, this disparity became more pronounced when contrasting the enriched expression in the testis and epididymis of all genes in branch 5 of the MR secretome with that of the remaining branch 5 genes (43/50 vs 12/192; 2TFET, P = 6.2×10^{-28}) (Fig. 3a).

4. Broader framing of epididymal secretions and paternal epigenetic inheritance. Beyond sperm function per se, the findings prompt consideration of emerging literature showing that epididymal secretions, particularly epididymsome cargo, contribute to shaping of the sperm epigenome and influencing offspring phenotypes. This post-testicular window has been proposed as a rapid and potentially adaptive interface between paternal environment and the next generation. While

speculative framing should remain measured, a brief acknowledgment of this literature could enhance the conceptual impact of the study. Relevant references include:

• Sharma et al., 2018, Developmental Cell

• Conine et al., 2018, Developmental Cell

• Trigg et al., 2019, Cell Reports

• Spadafora, 2023, Progress in Biophysics and Molecular Biology

• Trigg & Schjenken et al., 2024, PNAS

• Skerrett-Byrne et al., 2025, Molecular Nutrition & Food Research

This addition would help situate the evolutionary expansion of the epididymal secretome within a modern framework of paternal effects and intergenerational biology.

We acknowledge the relevant role of the epididymis in paternal epigenetic inheritance in lines 212-217, where the text reads:

"In eutherians, the epididymis is where complex sperm maturation occurs, including acrosomal matrix reorganization, sperm head rigidification, modifications of the sperm plasmalemma that mediate the initiation of sperm-zona binding, and shaping of the sperm epigenome, with the consequent potential alteration of paternal transgenerational effects (Bedford, 2014; Björkgren & Sipilä, 2019; Chan et al., 2020; Nixon et al., 2019b; Skerrett-Byrne et al, 2025)."

Referee #3:

This manuscript investigates the evolutionary dynamics of the human secretome by integrating Human Protein Atlas (HPA) annotations with vertebrate gene-age datasets (Chen et al. 2025 and GenTree). The authors report a punctuated expansion of the male reproductive (MR) secretome at the eutherian branch (Branch 5, Figs. 1, 2), specifically linked to epididymal enrichment and post-mating functions (Fig. 3). The study proposes that the epididymis acted as a "maturation laboratory" for sperm during the radiation of placental mammals, shifting from structural roles to regulatory and immune-related functions.

The conclusion that the epididymal/MR secretome underwent a major expansion during placental mammal diversification is novel and compelling, and aligns with current models of reproductive systems as hotspots for adaptive innovation. The integration of gene dating data with expression profile is methodologically sound, and routinely performed in the field of new gene origination. Thus, in principle, I support the publication of this manuscript. However, I have a few major or minor comments, which serves to further improve the manuscript.

We really appreciate the positive reception of our findings by the reviewer.

Major concerns

1. Since marsupials also possess an epididymis and undergo sperm maturation, I suggest that the authors discuss or analyze whether the eutherian secretome contains specific functional classes that are truly absent in theria (Branch 4), and whether these may be linked to placental-specific selective pressures. For example, why did particular glutathione peroxidases such as GPX5 and GPX6 evolve only in eutherians? Alternatively, could the expansion of the MR secretome have occurred independently in the marsupial lineage?

This is an interesting observation and we thank the reviewer from bringing it up to our attention. We have conducted a functional class enrichment for the genes that originated during theria radiation (branch 4) following the same approach we used for the analysis of MRS branch 5 (eutheria) vs the rest. We found only enrichment for three GO terms (single fertilization, extracellular region and acrosomal vesicle)

**Term**	**Score**	**Fold-Enrichment**
Single fertilization	2.52	4.41
Extracellular region	4.05	1.92
Acrosomal vesicle	2.79	4.19

These three GO terms are shared with MRS eutheria and other branches (see figure 3b). The result suggests that the eutherian secretome contains functional classes that are truly specific to that radiation. This is perhaps not surprising given the massive increase in gene numbers during the eutheria radiation (72) compared to all other branches (fig 1b). We find no indication of an independent MR secretome expansion in the ancestor of the marsupials and the eutherians. However, we are reluctant to include this in the revised version given the small number of MRS

genes we can assign to the theria branch (14) compared to the striking increase (72) in the eutheria branch.

To sum up, we do not find clear evidence of MR secretome expansion in theria, which might be partially the result of the limited number of MR secretome genes in that branch. Additionally, MR secretome-encoding genes originating in branch 5 do show functional enriched terms that do not appear in the analysis of the rest of the MR secretome (Fig. 3b).

2. In line 101, the authors state that the expansion is independent of multigene family proliferation. I am curious how the MR secretome at Branch 5 arises under this scenario. Do these genes represent potential de novo origins (i.e., genes without paralogs predating Branch 5)? If so, how many such genes are there?

The reviewer points to a very exciting avenue of future research: the origin of the genes across the MR secretome, and particularly to those at branch 5. In supplementary text S1, we have tried to provide additional context. Essentially, in that supplementary text we document:

- 50% (i.e. 36) have no reliable paralog according to DIOPT.
- For the other 50%, i.e. those with a reliable paralog, we find different patterns:

a) 10 have paralogs with other MR secretome-encoding genes originated during the eutherian radiation, forming 5 paralogroups;

b) the other 26 belong to paralogroups in which it is possible to find other genes (21) unrelated to MR secretome by branch 5 combination.

Examining in detail the relationship between the branch of origin of the 26 MR secretome genes and that of the other 21 external paralogs, only for 7 of the former is possible to find an external paralog originated prior to branch 5. In other words, for a very limited fraction of the 72 genes, there is some indication of a possible duplication event scenario. For the rest, *de novo* evolution cannot be discarded, but as we say at the end of the Sup. Text S1, a most parsimonious explanation exists: sequence similarity with other more ancient paralogs has been eroded, complicating the reconstruction of the events that accompanied their origin.

This issue warrants a thorough and extensive analysis that falls beyond the scope of this paper.

Minor concerns

1. The study relies on human gene age and expression data to define "epididymal enrichment" in eutherian mammals. The manuscript would be substantially strengthened if the authors could reproduce this pattern using gene age and expression data from another mammalian lineage (e.g., mouse). While GenTree provides mouse gene age data, I am unsure whether comparable HPA-like expression datasets are available for mouse. If such data are not readily accessible, or if the required analyses would be overly time-consuming, the authors could at least discuss the possibility of testing this pattern in other mammalian lineages in future work.

We agree that this would be the ideal situation but to the best of our knowledge there is no equivalent functional expression dataset in other vertebrates, including mouse. We acknowledge the need of such data in the closing paragraph of the paper (lines 221-225):

"*Nevertheless, a detailed understanding about the evolutionary sequence of molecular changes underlying such overhaul, as well as commonalities and more lineage-restricted changes in epididymis function, will only be possible once systematic transcriptomic and proteomic analyses of the epididymis are performed across the vertebrate phylogeny*."

2. Some typos are present in figures.

o Figure 2b Legend: Correction needed: "paralago" should be "paralog".

Corrected.

o Figure 3a: Please clarify why 176 testis-enriched genes are not classified within the MR secretome, and delete "Encoding" in the Y-axis label.

The reviewer has been misled by the erroneous wording used both in the main text and in the figure. The contrast performed involves the genes originated in branch 5 that are part of the MR secretome and the rest of the genes in that branch relative to preferential expression in the epididymis and testis. Our goal was to determine whether the association of enriched expression in epididymis is confined to branch 5 of the MR secretome-encoding genes or else it is a general property of all the genes that originated in branch 5. The result is clear: epididymis-preferential expression is significantly restricted to only MR secretome-encoding genes within branch 5. We have corrected the text, figure 3, and its corresponding legend accordingly. Further, "Encoding" has been removed as requested in the y-axis of the plot indicated by the reviewer.

o Figure 3c: In Y-axis legend, "Number"=>"Number of"

Corrected.

This is the new version of figure 3 with all the amendments:

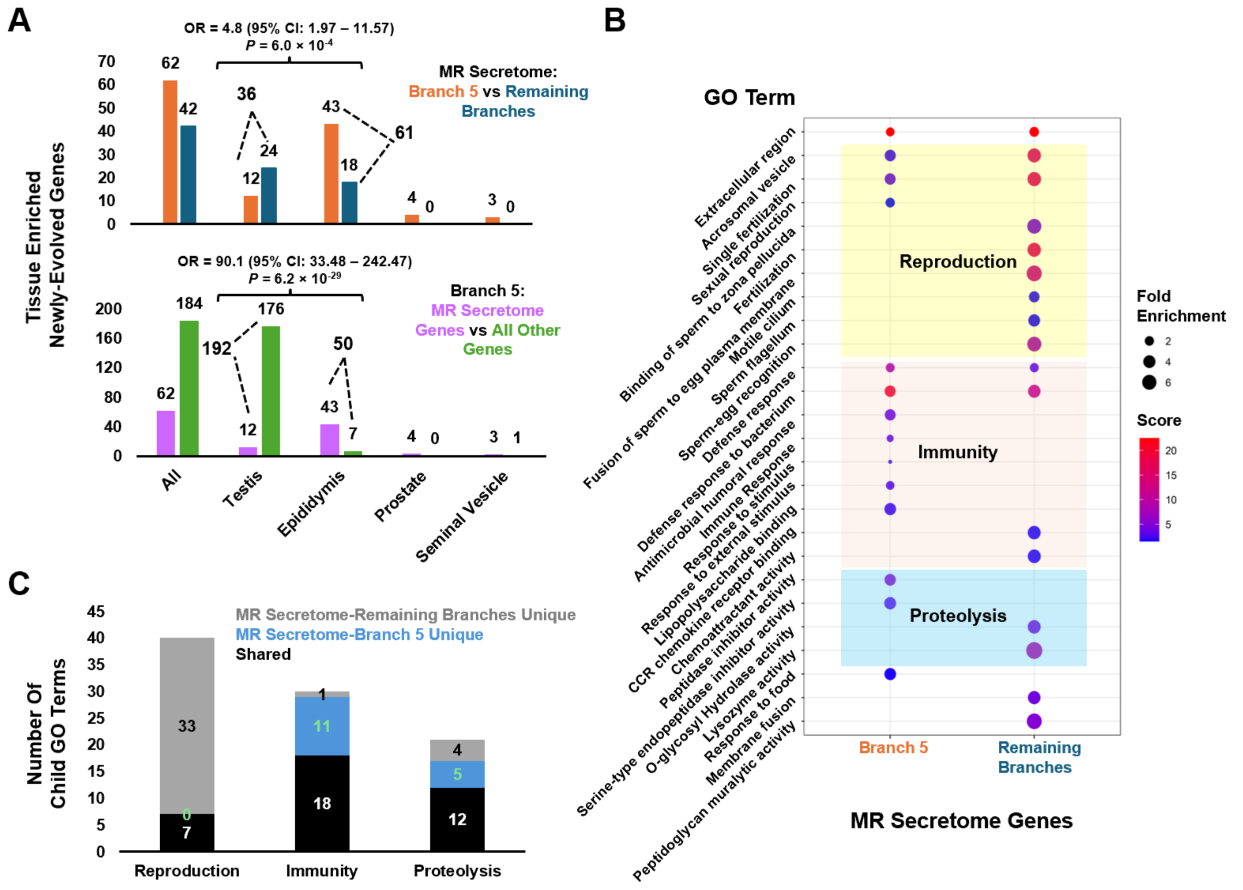

3. In line 74, the number 2510 appears inconsistent with the previously mentioned 2520. Please verify if this is a typo or the result of a specific filter.

There were 9 (not 10; we have corrected the inaccuracy so that it is now 2511) genes in the Human Proteome Atlas that we could not find in the list of genes provided in Chen et al. 2025. The reasons for this include the use of different annotation releases in Chen et al. 2025 and the secretome paper, which involved deprecated gene models. In the new submission, as part of the phylogenetic gene age data section, we have included this text (lines 242-244):

"*With the exception of nine secretome-encoding genes, all the rest were present in Chen et al. (Chen et al., 2025). Not present genes were associated with deprecated gene models in most recent gene annotation releases.*"

1st Revision - Editorial Decision 30th Apr 2026

Manuscript number: EMBOR-2026-63626V2
Title: Male reproductive biology was reshaped during placental mammal diversification through epididymal secretome expansion
Author(s): Jose Ranz and Alberto Civetta

Dear Prof. Ranz

Thank you for your patience while we have reviewed your revised manuscript. As you will see from the reports below, the referees are now all positive about its publication in EMBO reports. I am therefore writing with an 'accept in principle' decision, which means that I will be happy to accept your manuscript for publication once a few minor issues/corrections raised by our editorial assistance team have been addressed, as follows:

Data Availability statement: in, but needs to be renamed to Data Availability
COI/DCIS: in, but it needs to be renamed to Disclosure and Competing Interests Statement
Author contributions/CRediT: need to be removed from the manuscript text and included only in our submission system.
DATA NOT SHOWN: please see page 12. It is against our policy to allow such statements such as "Data not Shown". Please rephrase accordingly.
CHECKLIST: missing, please provide.
FIGURE CALLOUTS: S1 is an incorrect callout, looks like the authors are referring to Appendix figure S1, and we don't have it OR perhaps they are referring to Dataset S1 which is deposited at Dryad; "supporting information" should not be used as a callout (it is in the DAS, so the sentence needs updating anyway as we only need info on the deposited data in the DAS).
DATASET EV LEGENDS: not provided in our system, but there is Dataset S1 deposited at Dryad; the correct nomenclature should be Dataset EV1 (although this file is not located in our system nor it is called out in the manuscript text).
APPENDIX FILE WITH ToC: in, but also has some text labeled as Appendix texts S1 and Appendix Text S2 - we do not use this nomenclature so it should probably be removed from the table of contents and from the callouts in the manuscript (here it can just be called out as Appendix).
SYNOPSIS IMAGE: missing, please provide.
SYNOPSIS TEXT: missing, please provide.

- Figure Legends should be placed at the end of the manuscript text.

DATA CHECK:
Make sure that all relevant data deposited in Dryad is mentioned properly and is publicly available.

Figure Legends - Comments
- Please note that information related to n is missing in the legends of figures 2A, B. Please provide.
- Please note that the measure of center for the error bars needs to be defined in the legends of figures 2A, B. Please correct.

Once you have made these minor revisions, please use the following link to submit your corrected manuscript:

Link Not Available

If all remaining corrections have been attended to, you will then receive an official decision letter from the journal accepting your manuscript for publication in the next available issue of EMBO Reports. This letter will also include details of the further steps you need to take for the prompt inclusion of your manuscript in our next available issue.

Thank you for your contribution to EMBO Reports.

Yours sincerely,

Yehu Moran
Academic Editor
EMBO Reports

Referee #2:

The authors have sufficiently addressed all my concerns and am very happy to see it published soon.

Referee #3:

The authors have addressed my comments well and I recommend its publication.

All minor editorial requests have been addressed by the authors.

Prof. Jose Ranz
University of California-Irvine
Ecology and Evolutionary Biology
United States

Dear Prof. Ranz and Prof. Civetta,

I am very pleased to accept your manuscript for publication in the next available issue of EMBO reports. Thank you for your contribution to our journal.

Your manuscript will be processed for publication by EMBO Press. It will be copy edited and you will receive page proofs prior to publication. Please note that you will be contacted by Springer Nature Author Services to complete licensing and payment information.

You may qualify for financial assistance for your publication charges - either via a Springer Nature fully open access agreement or an EMBO initiative. Check your eligibility: https://link.springer.com/journal/44319/how-to-publish-with-us

Should you be planning a Press Release on your article, please notify the production team via this form in order to coordinate publication and release dates: https://forms.cloud.microsoft/e/eH4A8JVP6g

If you have any questions, please do not hesitate to contact the Editorial Office. Thank you for your contribution to EMBO Reports.

Yours sincerely,

Yehu Moran
Editor
EMBO Reports

>>> Please note that it is EMBO Reports policy for the transcript of the editorial process (containing referee reports and your response letter) to be published as an online supplement to each paper. If you do NOT want this, you will need to inform the Editorial Office via email immediately. More information is available here: https://link.springer.com/partners/embo-press/editorial-policies#Peer%20review

EMBO Press Author Checklist

Corresponding Author Name: Jose Ranz & Alberto Civetta
Journal Submitted to: EMBO Reports
Manuscript Number: EMBOR-2026-63626V3

USEFUL LINKS FOR COMPLETING THIS FORM
The EMBO Journal - Author Guidelines
EMBO Reports - Author Guidelines
Molecular Systems Biology - Author Guidelines
EMBO Molecular Medicine - Author Guidelines

Reporting Checklist for Life Science Articles (updated January
This checklist is adapted from Materials Design Analysis Reporting (MDAR) Checklist for Authors. MDAR establishes a minimum set of requirements in transparent reporting in the life sciences (see Statement of Task: 10.31222/osf.io/9sm4x). Please follow the journal's guidelines in preparing your
Please note that a copy of this checklist will be published alongside your article.

Abridged guidelines for figures
1. Data
The data shown in figures should satisfy the following conditions:
- the data were obtained and processed according to the field’s best practice and are presented to reflect the results of the experiments in an accurate and unbiased manner.
- ideally, figure panels should include only measurements that are directly comparable to each other and obtained with the same assay.
- plots include clearly labeled error bars for independent experiments and sample sizes. Unless justified, error bars should not be shown for technical
- if n<5, the individual data points from each experiment should be plotted. Any statistical test employed should be justified.
- Source Data should be included to report the data underlying figures according to the guidelines set out in the authorship guidelines on Data

2. Captions
Each figure caption should contain the following information, for each panel where they are relevant:
- a specification of the experimental system investigated (eg cell line, species name).
- the assay(s) and method(s) used to carry out the reported observations and measurements.
- an explicit mention of the biological and chemical entity(ies) that are being measured.
- an explicit mention of the biological and chemical entity(ies) that are altered/varied/perturbed in a controlled manner.
- the exact sample size (n) for each experimental group/condition, given as a number, not a range;
- a description of the sample collection allowing the reader to understand whether the samples represent technical or biological replicates (including how many animals, litters, cultures, etc.).
- a statement of how many times the experiment shown was independently replicated in the laboratory.
- definitions of statistical methods and measures:
  - common tests, such as t-test (please specify whether paired vs. unpaired), simple χ2 tests, Wilcoxon and Mann-Whitney tests, can be unambiguously identified by name only, but more complex techniques should be described in the methods section;
  - are tests one-sided or two-sided?
  - are there adjustments for multiple comparisons?
  - exact statistical test results, e.g., P values = x but not P values < x;
  - definition of ‘center values’ as median or average;
  - definition of error bars as s.d. or s.e.m.

Please complete ALL of the questions below.
Select "Not Applicable" only when the requested information is not relevant for your study.

Materials

Newly Created Materials	Information included in the manuscript?	In which section is the information available? (Reagents and Tools Table, Materials and Methods, Figures, Data Availability Section)
New materials and reagents need to be available; do any restrictions apply?	Not Applicable	Not Applicable

Antibodies	Information included in the manuscript?	In which section is the information available? (Reagents and Tools Table, Materials and Methods, Figures, Data Availability Section)
For **antibodies** provide the following information:<br>- Commercial antibodies: RRID (if possible) or supplier name, catalogue number and or/clone number<br>- Non-commercial: RRID or citation	Not Applicable	Not Applicable

DNA and RNA sequences	Information included in the manuscript?	In which section is the information available? (Reagents and Tools Table, Materials and Methods, Figures, Data Availability Section)
**Short novel DNA or RNA including primers, probes**: provide the sequences.	Not Applicable	Not Applicable

Cell materials	Information included in the manuscript?	In which section is the information available? (Reagents and Tools Table, Materials and Methods, Figures, Data Availability Section)
**Cell lines:** Provide species information, strain. Provide accession number in repository **OR** supplier name, catalog number, clone number, and/**OR** RRID.	Not Applicable	Not Applicable
**Primary cultures:** Provide species, strain, sex of origin, genetic modification status.	Not Applicable	Not Applicable
Report if the cell lines were recently **authenticated** (e.g., by STR profiling) and tested for mycoplasma contamination.	Not Applicable	Not Applicable

Experimental animals	Information included in the manuscript?	In which section is the information available? (Reagents and Tools Table, Materials and Methods, Figures, Data Availability Section)
**Laboratory animals or Model organisms:** Provide species, strain, sex, age, genetic modification status. Provide accession number in repository **OR** supplier name, catalog number, clone number, **OR** RRID.	Not Applicable	Not Applicable
**Animal observed in or captured from the field:** Provide species, sex, and age where possible.	Not Applicable	Not Applicable
Please detail **housing and husbandry conditions**.	Not Applicable	Not Applicable

Plants and microbes	Information included in the manuscript?	In which section is the information available? (Reagents and Tools Table, Materials and Methods, Figures, Data Availability Section)
**Plants:** provide species and strain, ecotype and cultivar where relevant, unique accession number if available, and source (including location for collected wild specimens).	Not Applicable	Not Applicable
**Microbes:** provide species and strain, unique accession number if available, and source.	Not Applicable	Not Applicable

Human research participants	Information included in the manuscript?	In which section is the information available? (Reagents and Tools Table, Materials and Methods, Figures, Data Availability Section)
If collected and within the bounds of privacy constraints report on age, sex and gender or ethnicity for all study participants.	Not Applicable	Not Applicable

Core facilities	Information included in the manuscript?	In which section is the information available? (Reagents and Tools Table, Materials and Methods, Figures, Data Availability Section)
If your work benefited from core facilities, was their service mentioned in the acknowledgments section?	Not Applicable	Not Applicable

Design

Study protocol	Information included in the manuscript?	In which section is the information available? (Reagents and Tools Table, Materials and Methods, Figures, Data Availability Section)
If study protocol has been **pre-registered, provide DOI in the manuscript**. For clinical trials, provide the trial registration number **OR** cite DOI.	Not Applicable	Not Applicable
Report the **clinical trial registration number** (at ClinicalTrials.gov or equivalent), where applicable.	Not Applicable	Not Applicable

Laboratory protocol	Information included in the manuscript?	In which section is the information available? (Reagents and Tools Table, Materials and Methods, Figures, Data Availability Section)
Provide DOI OR other citation details if **external detailed step-by-step protocols** are available.	Not Applicable	Not Applicable

Experimental study design and statistics	Information included in the manuscript?	In which section is the information available? (Reagents and Tools Table, Materials and Methods, Figures, Data Availability Section)
Include a statement about **sample size** estimate even if no statistical methods were used.	Yes	Figure legends & Methods
Were any steps taken to minimize the effects of subjective bias when allocating animals/samples to treatment (e.g. **randomization procedure**)? If yes, have they been described?	Not Applicable	Not Applicable
Include a statement about **blinding** even if no blinding was done.	Not Applicable	Not Applicable
Describe **inclusion/exclusion criteria** if samples or animals were excluded from the analysis. Were the criteria pre-established?<br><br>If sample or data points were omitted from analysis, report if this was due to attrition or intentional exclusion and provide justification.	Yes	Only 9 genes without gene age estimate were not considered in downstream analyses
For every figure, are **statistical tests** justified as appropriate? Do the data meet the assumptions of the tests (e.g., normal distribution)? Describe any methods used to assess it. Is there an estimate of variation within each group of data? Is the variance similar between the groups that are being statistically compared?	Yes	No parametric test was used

Sample definition and in-laboratory replication	Information included in the manuscript?	In which section is the information available? (Reagents and Tools Table, Materials and Methods, Figures, Data Availability Section)
In the figure legends: state number of times the experiment was **replicated** in laboratory.	Not Applicable	Not Applicable
In the figure legends: define whether data describe **technical or biological replicates**.	Not Applicable	Not Applicable

Ethics

Ethics	Information included in the manuscript?	In which section is the information available? (Reagents and Tools Table, Materials and Methods, Figures, Data Availability Section)
Studies involving **human participants**: State details of **authority granting ethics approval** (IRB or equivalent committee(s), provide reference number for approval.	Not Applicable	Not Applicable
Studies involving **human participants**: Include a statement confirming that **informed consent** was obtained from all subjects and that the experiments conformed to the principles set out in the WMA Declaration of Helsinki and the Department of Health and Human Services Belmont Report.	Not Applicable	Not Applicable
Studies involving **human participants:** For publication of **patient photos**, include a statement confirming that consent to publish was obtained.	Not Applicable	Not Applicable
Studies involving experimental **animals**: State details of **authority granting ethics approval** (IRB or equivalent committee(s), provide reference number for approval. Include a statement of compliance with ethical regulations.	Not Applicable	Not Applicable
Studies involving **specimen and field samples:** State if relevant **permits** obtained, provide details of authority approving study; if none were required, explain why.	Not Applicable	Not Applicable

Dual Use Research of Concern (DURC)	Information included in the manuscript?	In which section is the information available? (Reagents and Tools Table, Materials and Methods, Figures, Data Availability Section)
Could your study fall under dual use research restrictions? Please check biosecurity documents and list of **select agents and toxins** (CDC): https://www.selectagents.gov/sat/list.htm	Not Applicable	Not Applicable
If you used a select agent, is the security level of the lab appropriate and reported in the manuscript?	Not Applicable	Not Applicable
If a study is subject to dual use research of concern regulations, is the name of the **authority granting approval and reference number** for the regulatory approval provided in the manuscript?	Not Applicable	Not Applicable

Reporting

The MDAR framework recommends adoption of discipline-specific guidelines, established and endorsed through community initiatives. Journals have their own policy about requiring specific guidelines and recommendations to complement MDAR.

Adherence to community standards	Information included in the manuscript?	In which section is the information available? (Reagents and Tools Table, Materials and Methods, Figures, Data Availability Section)
State if relevant guidelines or checklists (e.g., **ICMJE, MIBBI, ARRIVE, PRISMA**) have been followed or provided.	Not Applicable	Not Applicable
For **tumor marker prognostic studies,** we recommend that you follow the **REMARK** reporting guidelines (see link list at top right). See author guidelines, under 'Reporting Guidelines'. Please confirm you have followed these guidelines.	Not Applicable	Not Applicable
For **phase II and III randomized controlled trials**, please refer to the **CONSORT** flow diagram (see link list at top right) and submit the CONSORT checklist (see link list at top right) with your submission. See author guidelines, under 'Reporting Guidelines'. Please confirm you have submitted this list.	Not Applicable	Not Applicable

Data Availability

Data availability	Information included in the manuscript?	In which section is the information available? (Reagents and Tools Table, Materials and Methods, Figures, Data Availability Section)
Have **primary datasets** been deposited according to the journal's guidelines (see 'Data Deposition' section) and the respective accession numbers provided in the Data Availability Section?	Not Applicable	Not Applicable
Were **human clinical and genomic datasets** deposited in a public access-controlled repository in accordance to ethical obligations to the patients and to the applicable consent agreement?	Not Applicable	Not Applicable
Are **computational models** that are central and integral to a study available without restrictions in a machine-readable form? Were the relevant accession numbers or links provided?	Not Applicable	Not Applicable
If publicly available data were reused, provide the respective **data citations in the reference list**.	Yes	Methods
